# The Andean Adaptive Toolkit to Counteract High Altitude Maladaptation: Genome-Wide and Phenotypic Analysis of the Collas

**DOI:** 10.1371/journal.pone.0093314

**Published:** 2014-03-31

**Authors:** Christina A. Eichstaedt, Tiago Antão, Luca Pagani, Alexia Cardona, Toomas Kivisild, Maru Mormina

**Affiliations:** 1 Division of Biological Anthropology, University of Cambridge, Cambridge, Cambridgeshire, United Kingdom; 2 Wellcome Trust Centre for Human Genetics, University of Oxford, Oxford, Oxfordshire, United Kingdom; 3 Wellcome Trust Sanger Institute, Hinxton, Cambridgeshire, United Kingdom; 4 School of Chemistry, University of East Anglia, Norwich, Norfolk, United Kingdom; Universitat Pompeu Fabra, Spain

## Abstract

During their migrations out of Africa, humans successfully colonised and adapted to a wide range of habitats, including extreme high altitude environments, where reduced atmospheric oxygen (hypoxia) imposes a number of physiological challenges. This study evaluates genetic and phenotypic variation in the Colla population living in the Argentinean Andes above 3500 m and compares it to the nearby lowland Wichí group in an attempt to pinpoint evolutionary mechanisms underlying adaptation to high altitude hypoxia. We genotyped 730,525 SNPs in 25 individuals from each population. In genome-wide scans of extended haplotype homozygosity Collas showed the strongest signal around *VEGFB*, which plays an essential role in the ischemic heart, and *ELTD1*, another gene crucial for heart development and prevention of cardiac hypertrophy. Moreover, pathway enrichment analysis showed an overrepresentation of pathways associated with cardiac morphology. Taken together, these findings suggest that Colla highlanders may have evolved a toolkit of adaptative mechanisms resulting in cardiac reinforcement, most likely to counteract the adverse effects of the permanently increased haematocrit and associated shear forces that characterise the Andean response to hypoxia. Regulation of cerebral vascular flow also appears to be part of the adaptive response in Collas. These findings are not only relevant to understand the evolution of hypoxia protection in high altitude populations but may also suggest new avenues for medical research into conditions where hypoxia constitutes a detrimental factor.

## Introduction

In the last 40,000 years modern humans have undergone a series of rapid adaptive changes in response to new environmental pressures as they spread from Africa into new habitats [1]. High altitude (HA) is one of the most extreme environments, characterised by low concentrations of atmospheric oxygen (hypoxia), wide temperature ranges and other concomitant environmental variables, resulting in significant physiological stress [2]. Yet, ca. 466 million people live permanently at altitudes above 3000 m [3]. Effective adaptive mechanisms are known to be in place to contend with the effects of chronic hypoxia. These are also known to differ and be convergent among the main HA populations: Tibetans, Andeans and Ethiopians [4]. Given the relatively recent time scale of peopling of the Himalayas and the Andes [5] these convergent patterns suggest strong selective pressures upon putative beneficial traits. As hypoxia is also a major factor in a number of pathologies [2], [6], HA populations represent an ideal natural experiment to understand the biology of the hypoxic response.

HA literature on Andean highlanders has focused so far on Aymara and Quechua groups [7]–[17]. Thus, studying a different HA population may allow us to test whether or not the same signatures of selection are present across the whole Andean range and to grasp the breadth of physiological and molecular responses at play during hypoxia.

In non-native highlanders the process of acclimatisation to HA triggers a number of rapid, short-term physiological responses, including increase in the basic metabolic rate (BMR) [18], rise in haematocrit via the upregulation of erythropoietin (EPO) synthesis and reduction of plasma volume [19], elevated ventilation rate [2] and secretion of vascular endothelial growth factor (VEGF) to allow better blood perfusion [20]. The high haematocrit increases blood viscosity and shear force in the blood vessels. If permanent, these effects can be maladaptive, as they intensify heart labour and can result in right ventricular hypertrophy over time, with increased risk of heart failure [21].

Despite these negative effects, the typical Andean adaptation to hypoxia does involve a permanently raised haematocrit [22]. Consequently, blood viscosity is well above the estimated optimal levels at HA [23], [24] and thus, in order to offset its maladaptive effects, Andeans have increased pulmonary vasoconstriction [11] to improve blood flow and arterial oxygen content [4], [22]. Furthermore, their pulmonary artery wall is protected by an additional layer of muscles [25], probably reducing the impact of increased lifelong pulmonary arterial blood pressure [26]. Other adaptations include thoracic expansion, increased lung capacities, a blunted hypoxic ventilatory response (HVR) [11], decreased cerebral blood flow velocity (CBFV) [27] and an almost normal respiration rate [7]. Recent studies on Tibetans highlighted the role of *EPAS1* and *EGLN1* genes in HA adaptation [15], [28]–[33]. The derived variants that are frequent in Tibetans are associated with reduced haemoglobin concentrations [33]–[35]. Interestingly, a signature of selection on *EGLN1* was also highlighted in Andeans [15] and in Daghestani from the Caucasus [36], though an association with haemoglobin concentrations could not be established in the former [37]. In Quechua and Aymara several other candidate genes have been detected by selection scans, including *SENP1* and *ANP32D* [17]. These show higher transcriptional activity in individuals with Chronic Mountain Sickness (CMS) [17] compared to healthy Andean controls, which probably contributes to the elevated haematocrit typical of this common maladaptation.

Among the genes identified as positively selected in Andeans by genotype based genome-wide scans are the transforming growth factor α (*TGFA*), the energy sensing kinase *PRKAA1* and the inducible neuronal NO synthases [15], [16]. Nitric oxide (NO) associated genes are also strong candidates for HA adaptation because NO production is elevated in Andeans, resulting in improved vasodilation and oxygen perfusion to tissues [38]. Another gene associated with the Andean adaptive response is the angiotensin converting enzyme (*ACE*), suggested to be at least partly responsible for Andean's close to normal levels of arterial oxygen saturation (SaO_2_) [12]. *ACE* is a key regulator of the renin-angiotensin-aldosterone system, a NO independent mechanism of blood pressure regulation. Finally, reactive oxygen species (ROS) genes are also potential candidates of selection. ROS formation is characteristic of oxidative stress and has been suggested to play a role in hypoxia signalling [39], though ROS excess damages the cell and can lead to apoptosis [40].

The above examples illustrate the complexity of the genetics of hypoxia adaptation. Genome-wide scans are powerful tools to identify signatures of selection but these approaches are known to produce false positives [41]. Thus, the validation of findings through cross verification from independent populations is essential. Our study not only offers cross-validation but also provides new insights into the Andean adaptive response.

The aim of our study was to assess genomic and phenotypic variation in the Colla group living above 3500 m in Northwest Argentina, and compare the detected signatures of selection to those previously reported in Aymara and Quechua. Contrary to our expectations, given the close ancestry of these three groups, we found population specific mechanisms and little overlap with previous studies. The two main candidate genes in Collas are associated with heart performance, one by increasing its vascularisation (*VEGFB*) and the other by regulating cardiac hypertrophy (*ELDT1*). This suggests an adaptive response to the lifelong pressure that a permanently elevated haematocrit imposes upon normal heart function.

## Materials and Methods

### Ethics statement and subjects

The study was approved by the Ethics Committee at the University of East Anglia, the Ministry of Health of the Province of Salta (Ministerio de Salud Pública, Salta, Argentina) and the University of Cambridge Human Biology Research Ethics Committee (HBREC.2011.01). Only healthy unrelated adults giving written informed consent were included in the study.

Individuals were sampled (Figure S1) at two different altitudes: Collas above 3500 m (high altitude, HA), and Wichí below 1000 m (low altitude, LA). We determined long-term residence by establishing that the birthplaces of parents and grandparents corresponded to the respective altitude, i.e. <1000 m for LA and >3000 m for HA. The Collas inhabit Northwest Argentina, Southern Bolivia and Northern Chile and are considered to be related to other Andean groups such as Quechua, Aymara, Atacameño, Omaguaca and possibly Diaguita [42].These groups could trace back to the beginning of human settlement in the Andes, which archaeological evidence places between 12,000 and 9,000 years before present [43]–[45].

Wichí live in a lowland area along the river Pilcomayo [42] known as the Gran Chaco, spanning Northeast Argentina, Bolivia and Paraguay, and likely originate from local hunter-gatherer groups [46]. They have continuously inhabited the region for 4,000–5,000 years [47]. This group was deemed to be more appropriate as a lowland control population than Amerindians currently represented in SNP panels such as HGDP because of their more recent shared ancestry with Collas, yet sufficiently differentiated in terms of language, culture and subsistence strategies. They also have low levels of possible confounding European admixture [48]–[50].

A brief interview was carried out to establish age, known medical conditions, as well as smoking behaviour. The time and type of the last meal before sampling were also recorded. Meal contents were subsequently translated into caloric intake using the national nutrient programme USDA Food Search for Windows Version 1.0, database version SR21 [51] and added as a variable to the dataset, in order to account for the effects of post-prandial hypotension on the physiological measurements taken.

### Genetic analyses

Saliva samples were collected [52] and DNA extracted according to published protocols (Qiagen DNA Investigator Kit). For each population, 25 samples were genotyped using the Illumina HumanOmniExpress BeadChip for 730,525 SNPs. Only samples and SNPs with genotype call rate of >98% were included in downstream analyses, with 726,090 SNPs meeting this requirement. Genotype data were phased together with HapMap 3 data using SHAPEIT [53]. Five samples were excluded because they either did not pass the identity by descent (IBD) criterion of <0.125 or had high percentage of European admixture, resulting in final dataset of 20 Wichí and 23 Collas. The data has been deposited with NCBI GEO (accession numbers GSM1330751-GSM1330801).

Mitochondrial haplogroups were determined by sequencing the hypervariable region I (HV I) between positions 15908 and 16498 using published PCR protocols and primers [54], [55]. Haplogroup diversity of mitochondrial sequences was assessed with the θ(π) [56] and Nei's genetic diversity estimate [57] using Arlequin 3.5.3.1 [58].

Restriction fragment length polymorphisms (RFLP) were used to assess Y-chromosome haplogroups (Table S1). All samples were screened for the most common South American haplogroup Q and the most prevalent European haplogroup R1b based on previously reported frequencies in the Argentinean population [59]. In case of non-assigned samples further sequencing of Y-chromosome haplogroups would have been carried out.

### Demographic analyses

SNPs in LD (r^2^>0.1) were removed with PLINK (–indep-pairwise 50 10 0.1) [60] and a set of overlapping SNPs with 90% genotyping rate of SNPs across samples (–geno 0.1) was determined combining our data set with three HapMap populations [61] (Yoruba [YRI], Han Chinese from Beijing [CHB] and Utah residents with ancestry from northern and western Europe [CEU]), four populations from the Human Genome Diversity Project (HGDP) [62] (Karitiana, Suruí, Pima and Piapoco), Aymara and Quechua populations [63] and additional 13 Native American populations [64]. This resulted in 16,574 SNPs for subsequent analyses. The programme ADMIXTURE [65] was used to generate admixture proportions and was run 100 times for K values 2–10. The best value of K for all runs was determined by cross-validation (CV) and log-likelihood estimates [66]. The log-likelihood difference between minimum and maximum of each K was calculated. Principal component analysis (PCA) was performed using SmartPCA implemented in the EIGENSOFT package [67]. Migration events among populations were inferred with the programme TreeMix [68] on 178,076 overlapping SNPs with 90% genotyping rate. Windows of 600 SNPs were chosen to obtain approximately 10 Mb blocks. HapMap Yoruba (YRI) was specified as outgroup and 100 bootstrap replicates were generated to produce a consensus tree. The *f4* statistic [69] was used to independently assess the support for suggested migrations.

### Phenotypic measurements

Oxygen saturation (SaO_2_) and heart rate (HR) at rest were measured simultaneously with a Digital Pulse Oximeter (model 8500, Nonin Medical Inc, USA) with values not visible to participants [70]. Respiratory rate at rest was determined by the counting method. Anthropometric measurements were obtained following Frisancho [71] and Cameron *et al* [72]. These included: height (Leicester Height Measure, Seca, UK), weight (Body Composition Meter BC-520, Tanita, USA), and chest breadth and chest depth (Harpenden anthropometer, Holtain, UK). Chest extensions were measured at the height of the fifth thoracic vertebra during tidal breathing at maximum expiration and inhalation and averaged. Due to a highly skewed distribution a log-transformation was chosen and 0.05 added to avoid the logarithm of zero.

Weight, body fat, visceral fat and basic metabolic rate (BMR) were recorded with a bio-impedance scale, based on height, age, gender and fitness (determined as >10 h of sport/week). We calculated BMI as weight/(height)^2^ and measured diastolic (BP_DIAS_) and systolic (BP_SYS_) blood using a wrist monitor placed on the left arm (SBC 28, Sanitas, Germany) as the average of three measurements. Cardiac output (CO) was roughly estimated considering constant arterial stiffness and a stroke volume to pulse pressure (PP) relationship equivalent to that measured in healthy subjects [73]. The following formula was used: CO = PP*1.49*HR, where (PP) = BP_SYS_-BP_DIAS_.

The vascularisation of the face was measured with a reflectometer (DermaSpectrometer, DSM II Color Meter, Cortex Technology, Denmark). Measurements were taken 2 cm below the centre of the left eye. The melanin index, as well as a* (red-green axis) and L*-values (lightness-darkness axis) were recorded.

The Statistical Programme for Social Sciences (SPSS) V. 21 was used for the statistical analysis of phenotypic measurements. An independent t-test was applied to compare phenotypic differences between populations. If variances were not equal (Levene's Test was significant) a corrected t-value was considered. To confirm that significant differences between populations were due to altitude and not confounded e.g. by age or gender, a general linear model (GLM) type I was used.

### Tests for positive selection

The integrated haplotype score (iHS) and cross population extended haplotype homozygosity test (XP-EHH) were implemented as in Pickrell *et al* [74]. Genetic distances between SNPs were calculated from the HapMap genetic map [75]. Ancestral and derived states for each site were taken from the Ensembl Variation Database Release 68 [76]. Bins were created according to the number of SNPs located within a window. Four bins (20–39, 40–59, 60–79 and ≥80 SNPs) were used in the assessment of empirical p-values for iHS and five for XP-EHH (additional bin <19 SNPs). A cut-off of 1% was used and any genes present in the 5% top end of the iHS distribution of Wichí were excluded from iHS Colla results.

Pairwise *F*_ST_ between HA and LA populations were calculated using the programme GENEPOP [77], [78]. We recorded maximum *F*_ST_ values per 200 kb window in the top 1%. The population branch statistic (PBS) was estimated [33], [79] for Collas using Wichí and Siberian Eskimos as reference groups (A.C. unpublished data). Eskimos were chosen as the closest non-American outgroup genotyped on the same genotyping platform as Collas and Wichí. PBS was calculated for 100 kb windows, using a modified approach from Pickrell *et al* [74]. Windows were ranked by maximum PBS score.

We determined an *a priori* gene list to analyse the top 1% hits of the four selection tests in order to identify genes closely implicated in hypoxia response. The list consisted of five different pathways and 213 non-overlapping genes (see Table 1 and Table S2 for a detailed list of genes).

**Table 1:** Pathways determining hypoxia candidate genes for this study.

Hypoxia associated gene annotation	Reason for pathway choice	Identifier	Genes
GO term: “Cellular response to hypoxia”	More concise list than the general GO term “Hypoxia response” (225 genes)	GO:0071456	88
GO term: “Cellular response to ROS”	ROS formation is characteristic of oxidative stress	GO:0034614	73
REACTOME pathway: “NO stimulates guanylate cyclase”	Includes all genes involved in NO induced vasodilation	REACT_23862.1	28
REACTOME pathway: “Metabolism of angiotensinogen to angiotensins”	Renin-angiotensin-aldosterone system is a key mechanism for blood pressure regulation	REACT_147707.2	14
REACTOME pathway: “Signalling by VEGF”	Allows differential evaluation of vascularisation in Andeans	REACT_12529.1	10

### Enrichment analyses

We scanned windows in the top 1% of the iHS and XP-EHH distributions for enrichment of Gene Ontology (GO) terms. GO terms that appeared twice or more in any given window were considered only once in the analyses. A list of all genes in the top 1% windows was obtained using the Expression Analysis Systematic Explorer (EASE) score p-value implemented in DAVID [80]. GO terms were considered significantly enriched if the EASE-score was ≤0.01. Since PBS is an allele specific test, genes mapping to the SNP exhibiting the maximum PBS value in each window in the top 1% were used as an input into DAVID to evaluate gene enrichment [81], [82].

### Haplotype length and age estimation

To estimate the age of a haplotype, haplotype length was measured by extended haplotype homozygosity (EHH) [83]. It describes the probability that two sequences drawn from a given gene pool are homozygous from a defined base pair to a core SNP [83]. We calculated EHH for high ranking regions identified by XP-EHH and iHS starting from a core SNP with the highest derived iHS or XP-EHH value. An EHH value of 0.3 was considered as threshold, adapted from Voight *et al* [84]. The estimated EHH-length was then used to calculate the age of the haplotype [84] assuming a human generation time of 29 years [85]: P(Homozygosity) = e^−2RG^, R = Haplotype extent in cM, G = Generation time.

## Results

### Population differentiation at high and low altitude

**Figure 1:**
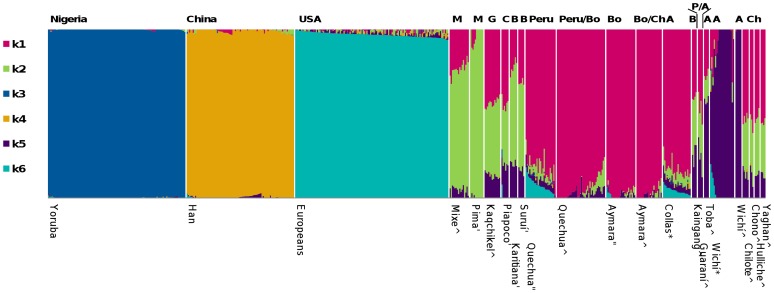
ADMIXTURE components of Argentinean Natives in a worldwide context. Populations are divided by six admixture proportions as K = 6 indicated the best fit for the data. The main proportions are derived from Yoruba (k3), Han Chinese (k4), Europeans (k6), Mexicans (Mixe/Pima, k2), Andean populations (k1) and Wichí (k5). Collas are indistinguishable from Aymara and Quechua, while Chilean Andeans mainly consist of Andean (k1) and Mixe/Pima (k2) characteristic admixture proportions. Gran Chaco populations (Kaingang, Chané, Guaraní and Toba) carry Wichí specific admixture proportions among others. The population name is displayed underneath the admixture plot while the sample origin is listed above (A: Argentina, B: Brazil, Bo: Bolivia, C: Colombia, Ch: Chile, G: Guatemala, M: Mexico, P:Paraguay) The population name is followed by a sign designating its study (°: HapMap, ∧: Reich *et al* [64], ‘: HGDP, “: Mao *et al* [63], *: this study).

Colla highlanders were compared to lowland Wichí, other Native American [62]–[64] and Chinese, European and African populations [61]. ADMIXTURE analyses showed similar ancestry components in Collas, Aymara and Quechua at K = 6 (Figure 1). Wichí and other Gran Chaco populations shared an ancestry component that is uncommon in highland populations. European admixture proportions were low at K = 6, with 4% on average in Collas and 2% in Wichí. At K = 6 mean CV-error across 100 iterations was the lowest (Figure S2) and log-likelihood estimates varied the least at K = 6 compared to K = 5 and K = 7 (Figure S3), indicating the best match for the data (see Figure S4 for K = 2 to K = 5 results).

**Figure 2:**
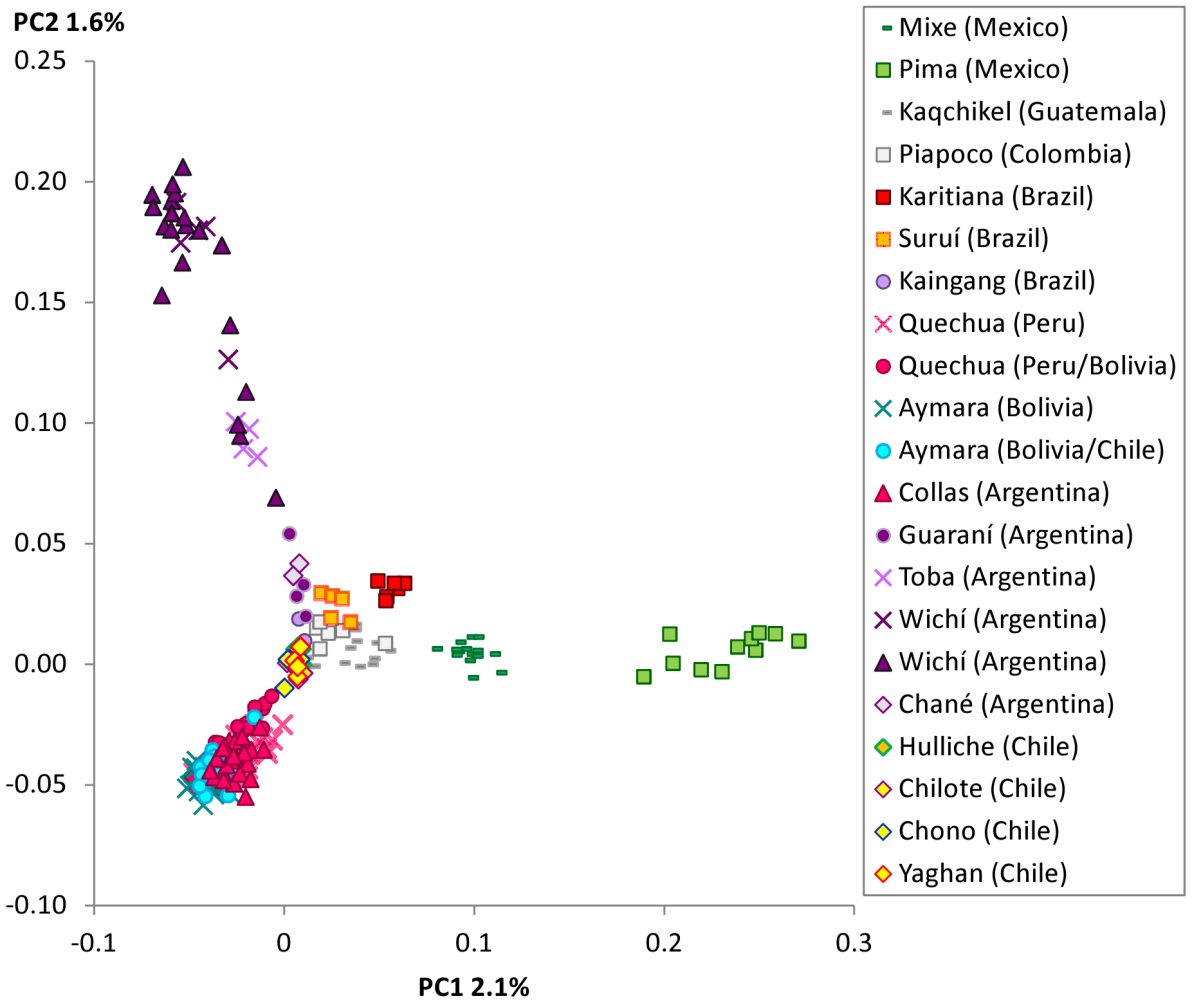
PCA of Native Americans from Mexico to Chile. Triangles: populations of this study; squares: HGDP, Quechua (Peru) and Aymara (Bolivia) were first published by Mao *et al* [63], remaining populations by Reich *et al* [64]. Wichí were included from this study and Reich *et al* [64] and Quechua and Aymara were both published by Mao *et al* [63] and Reich *et al* [64]. PC2 separates Wichí from Andean highland populations (Collas, Quechua and Aymara). PC1 distinguishes Mexican Pima and Mixe from the remaining populations. Collas cluster among Aymara and Quechua. The next closest populations are Chileans also from the Andean language family (Hulliche, Chilote, Chono and Yaghan). Gran Chaco populations (Wichí, Chané, Guaraní, Toba and Kaingang) show the widest spread while Wichí are as distinct to the Andean populations as Pima from Mexico.

To further assess genetic differentiation of Andeans from other Central and South American populations we performed PCA. Consistent with ADMIXTURE results, Collas clustered tightly with Quechua and Aymara while Wichí were outliers, clustering loosely with Toba and other Gran Chaco populations (Figure 2).

The impact of recent European admixture in Collas and Wichí was further assessed by analysing mitochondrial DNA (mtDNA) and Y-chromosome haplogroup frequencies. Overall, mitochondrial haplogroup diversity in Collas and Wichí was low, consistent with the general pattern across Native American populations (Table S3). All mitochondrial haplotypes clustered within Native American specific haplogroups (Table 2 and Table S4), whilst most Y-chromosome haplotypes clustered within Native American specific haplogroup Q, though the common European haplogroup R1b was also present in both populations.

**Table 2:** European admixture proportions in the two study populations.

Population	mtDNA		Y-chromosome^a^		autosomes	
	NA	EU	NA	EU	NA	EU
**Collas**	100%	0%	80%	20%	96%	4%
**Wichí**	100%	0%	90%	10%	98%	2%

NA: Native American specific haplogroups, EU: European specific haplogroups.

a 10 Colla men and 10 Wichí men were included.

**Figure 3:**
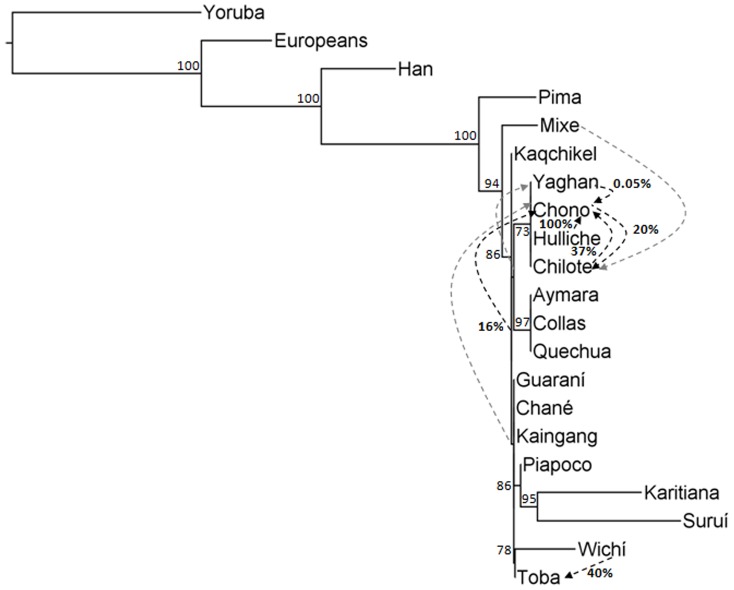
Consensus maximum likelihood tree for a reduced number of populations with 10 migration events. Non-bold numbers are bootstrap estimates based on 100 iterations with a support greater than 70%. Quechua, Aymara and Collas form one clade and group with Chilean Andean speakers (Yaghan, Hulliche, Chono and Chilote). Gran Chaco populations (Toba, Wichí, Guaraní, Chané and Kaingang) form a clade with Brazilians (Suruí and Karitiana) and Colombians (Piapoco). Mixe and Pima from Mexico cluster outside all South Americans and Kaqchikel from Guatemala. Branch length refers to the amount of drift experienced but is also increased in populations with more individuals in the data set. Black arrows indicate migrations confirmed as significant by *f*4 test, while grey arrows indicate insignificant *f*4 results. Bold numbers represent admixture proportions for black arrows: Toba received 40% admixture proportion from Wichí. Gene flow among Chilean Andeans was strongly supported: Hulliche contributed 100% admixture to Chono and HA Andeans 16%. An ancestral population of Chilote and Chono contributed 37% to Chono and 20% to Chilote. Yaghan contributed 0.05% admixture proportion to Chono.

We used genome-wide data from 18 Amerindian populations to build a phylogenetic ML tree (Figure 3). In agreement with our PCA, Collas, Aymara and Quechua formed a single clade, confirming their genetic relatedness. A close relationship between all Gran Chaco populations, including Wichí, was also confirmed. TreeMix analyses inferred gene flow from Wichí to Toba and multiple admixture events in Southern Chilean populations (Figure 3).

### Phenotypic comparisons between Collas and Wichí

The main phenotypic differences between Collas and Wichí are summarised in Table 3. Both groups differed significantly in their oxygen saturation (SaO_2_), and Wichí showed highest values for weight, BMI, systolic blood pressure and cardiac output. In contrast, thorax movement during breathing was greater in Colla, though thorax breadth and depth measurements themselves were not significantly different. These results suggest that either Collas do not have the typically enlarged Andean chest or that this trait is larger than expected in Wichí. The latter seems more plausible, as the chest measurements of Collas are comparable to those of Aymara and Quechua [7], [86], [87].

**Table 3:** Comparison of variables between Collas and Wichí.

Variable	Collas ± SD	Wichí ± SD
Oxygen saturation (%)	88.6±2.5	97.2±1.4^a^
Age (years)	40.4±12.8	42.2±13.0
Height (cm)	159.8±8.6	160.9±8.1
Weight (kg)	66.5±13.4	77.9±16.6^b^^, ^ c
Body fat (%)	26.1±12.6	31.45±10.1
Visceral fat	7.1±4.9	9.5±4.3
BMR (kcal)	1458.9±271.8	1595.5±354.4
BMI (kg/m^2^)	25.8±3.6	30.1±6.0^b^^, ^ c
Heart rate (1/min)	68.6±9.2	74.9±12.0
Systolic blood pressure (mmHg)	114.0±13.2	132.0±28.3^b^^, ^ c
Diastolic blood pressure (mmHg)	73.7±8.9	81.5±17.6
Cardiac output (ml/min)	4108.3±803.0	5692.8±2250.9^b^^, ^ c
Respiration rate (1/min)	20.2±4.5	17.9±3.9
Thorax breadth (cm)	30.4±3.0	31.4±2.8
Thorax depth (cm)	19.0±2.2	20.1±1.9
Log (Change in thorax breadth)	0.5±0.4	0.2±0.2^a^^, ^ c
Log (Change in thorax depth)	0.3±0.2	0.1±0.1^a^^, ^ c
a* (red green axis)	19.9±2.1	18.9±2.0
L* (lightness index)	17.9±3.0	16.9±1.7
Melanin index	57.0±4.9	56.8±2.3

*values correspond to the Commission Internationald'Eclairage L*a*b* system.

a p≤0.001,

b p<0.05.

c Equally significant after correction for further independent variables:

Weight: corrected for height, age and gender; BMI: corrected for age and gender;

Systolic blood pressure/Cardiac output: corrected for time since last meal, its caloric amount, age and gender;

Log (Change in thorax breadth/depth): corrected for age and gender.

### Identification of genes under positive selection

**Figure 4:**
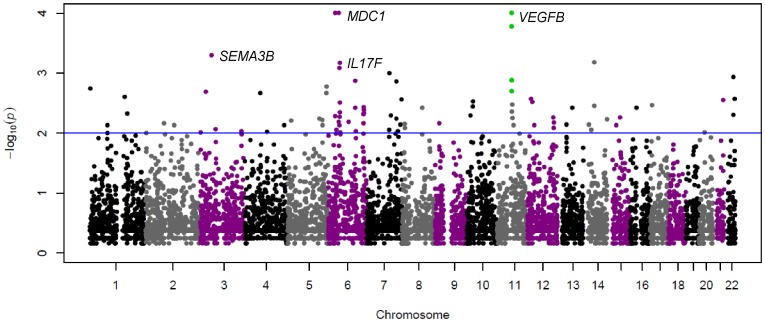
Manhattan plot of iHS window p-values across all chromosomes in Collas. The y-axis denotes the empirical p-value of the windows. The blue line indicates the 1% cut off. 12 windows in the top 1% of Collas were excluded since they overlapped with the top 5% of Wichí iHS windows. The highest empirical p-values in each bin were 0 and thus arbitrarily set to 0.001 to display them as highest values as the calculation of log_10_ (0) is not permitted. Chromosome 11 harbours the top window, with the highest p-value and greatest bin-score containing *VEGFB*; windows ±1 MB in this region are highlighted in green. The highest ranking window in the bin containing >80 SNPs included *MDC1*, a gene controlling DNA repair in response to hypoxia. The top window of the bin with 60–79 SNPs, which is located 16 Mb downstream from *MDC1*, did not contain plausible candidate genes. *SEMA3B* is involved in neuron development and *IL17F* in can inhibit angiogenesis. The highest window on chromosome 14 did not contain any genes.

We employed four selection tests to compare Collas to Native American lowlanders. The top 1% ranking iHS windows are reported in Figure 4 and Table S5. Twelve windows were excluded as they were also among the top 5% of iHS windows in Wichí. The topmost iHS window (Chr 11: 64–64.2 Mb) was found within a cluster of high ranking windows (Figure S5 and Table S6). Among the genes present in this region, three genes (*VEGFB*, *BAD* and *PRDX5*) from the *a priori* hypoxia candidate gene list (Table 1 and Table S2) mapped to the topmost window (Table 4). We calculated extended haplotype homozygosity (EHH) probability to assess the length of the haplotype around the Chr 11: 64–64.2 Mb locus [83]. This approach estimated an overall haplotype length of 1.4 Mb (0.998 cM) in Collas, extending 656 kb upstream and 785 kb downstream from the core SNP. This represents approximately twice the length of the same haplotype in Wichí (Table 5). We estimated the age of the haplotype [84] in Collas to be 3500 years.

**Table 4:** Hypoxia candidate genes in the 1% of iHS and XP-EHH results in Collas.

Test	Rank	Gene	Name	Function	Hypoxia association
**iHS**	1	*VEGFB*	Vascular endothelial growth factor β	Growth factor for endothelial cells, predominantly expressed in the ischemic heart	VEGF signalling
	1	*BAD*	BLC2-associted agonist of cell death	Positive regulation of cell apoptosis, hypoxia responsive	Cellular response to ROS
	1	*PRDX5*	Peroxisomal antioxidant enzyme	Reduces hydrogen peroxide	Cellular response to hypoxia
	15	*TP53*	Tumour protein p53	Binding partner of HIF-1, mediates cellular responses to DNA damage and hypoxia	Cellular response to hypoxia
	16	*STC2*	Stanniocalcin 2	Glycoprotein, essential for bone and skeletal muscle growth; HIF-1 activated	Cellular response to hypoxia
	72	*PDE2A*	Phosphodiesterase 2A	Signalling in vascular smooth muscle proliferation and contraction, cardiac contractility and platelet aggregation	NO stimulates guanylate cyclase
**XP-EHH**	10	*IL18BP*	Interleukin-18-binding protein	Inhibits interleukin 18, inferred involvement in cellular response to hydrogen peroxide	Cellular response to ROS
	102	*CCS*	Copper chaperone for superoxide dismutase	Stabilises and activates superoxide dismutase	Cellular response to ROS

**Table 5:** *VEGFB* haplotype characterised by EHH >0.3 in study populations.

Population	Haplotype Length (bp)	Homozygotes	Heterozygotes	Non-carriers
Collas	1,441,291	3 (13%)	9 (39%)	11 (48%)
Wichí	716,297	1 (5%)	4 (20%)	15 (75%)

We also screened the remainder of the top 1% scoring iHS windows against the *a priori* candidate gene list. We found three additional genes (*STC2*, *TP53* and *PDE2A*), two of which (*STC2* and *TP53*) are involved in cellular hypoxia responses and one in the NO pathway (*PDE2A*, see Table 4).

XP-EHH scores were determined in Collas using Wichí as a reference population [88]. Only two genes (*IL18BP* and *CCS*) from the *a priori* candidate gene list were found in the top 1% results of XP-EHH (Table 4 and Table S5). Both genes are involved in the detoxification of ROS in the cell. Besides mapping genes onto the *a priori* hypoxia candidate gene list, we also screened the top window in each of the five bins for other related genes that could be associated with HA adaptation. The two highest scoring windows in terms of p-value and bin-score contained *ELTD1*. This gene is essential for cardiac development and regulates cardiomyocyte growth and proliferation in the adult heart [89].

We also performed two allele frequency tests, pairwise *F*_ST_ and population branch statistic (PBS). Both search for unusually high allele frequency differentiation among populations. None of the genes from the *a priori* candidate gene list had unusually high pairwise *F*_ST_. While, the top *F*_ST_ window contained the calcium channel *KCNN2*, which is up-regulated under acute hypoxia [90], the SNP with the highest scoring *F*_ST_ value lies 91 kb upstream of the gene itself. Hence, we cannot establish unequivocally that the signal is driven by *KCNN2*, though it could be driven by an enhancer.

Seven genes among the top 1% PBS windows matched the hypoxia candidate gene list (Table S7). Four of these were associated with the GO term ‘cellular response to hypoxia’, two with ‘cellular response to ROS’ and one was part of the NO pathway. The second highest scoring window of PBS contained the *CBS* gene involved in cerebral blood flow regulation [91].

Though the four selection tests implemented in this study aimed to reveal different properties of the data and are not necessarily expected to identify the same genes, a total of 108 genes were highlighted by at least two statistics (see Table S8 for a list of all genes). Of these, only *STC2*, which is HIF activated and protects cells from apoptosis during hypoxia, matched the *a priori* hypoxia candidate gene list.

### Functional assessment of genes

The genes found in the top 1% windows of iHS, XP-EHH and PBS were used as an input list for GO term enrichment analysis. We did not find an overrepresentation of the HIF pathway. However, GO term analysis of iHS top 1% genes revealed 114 significantly enriched terms (EASE-score <0.01, Table S9), including the terms ‘cardiac ventricle formation’ and ‘cardiac chamber formation’ among the 15 most significant terms.

In addition to the iHS signal around *PDE2A*, the enrichment of three pathways involved in the regulation or formation of NO metabolites (Table S9) further suggests that NO-induced vasodilation is an important element of the Andean response to hypoxia. We also found enrichment of the categories ‘response to oxidative stress’, ‘response to reactive oxygen species’ and a number of pathways involved in DNA damage repair (Table S9).

The GO term enrichment of XP-EHH top 1% genes revealed 13 terms with an EASE-score <0.01 (Table S10). These terms were mainly related to general cell functions and neuron development. Enrichment analysis of the top 1% PBS genes resulted in 11 GO terms mainly related to ion transport and also neuron development (Table S11).

We investigated the haplotypes around the top iHS and XP-EHH candidate genes to assess possible phenotype-genotype correlations. Three Colla individuals were homozygous and nine heterozygous for the *VEGFB* haplotype defined by EHH = 0.3 (Table 5). We pooled together homozygotes and heterozygotes as ‘haplotype carriers’ assuming a dominant effect for the putative causative mutation; we also repeated the analysis with heterozygotes and homozygotes considered separately assuming a recessive model. Correlations between the presence or absence of the haplotype and likely related phenotypic traits were assessed using a general linear model (GLM). We did not find a significant correlation of the haplotype with oxygen saturation, blood pressure or any respiratory traits, neither under the recessive nor the dominant models (p>0.05, data not shown). Similarly, we found no genotype-phenotype correlation between *ELTD1* and either blood pressure, cardiac output, SaO_2_ or heart rate (p>0.05, data not shown).

## Discussion

To date the vast majority of HA studies have focused mainly on Tibetans [15], [28]–[33]; less research has been conducted on the other two major HA areas. It is only very recently that Ethiopians highlanders were included in genomic HA studies [92]–[94] and only three published genome-wide studies in Andeans are currently available [15]–[17], all including Quechua or Aymara populations. The Colla group chosen for this study is a HA population with recent shared ancestry to Aymara and Quechua, yet with sufficient degree of geographic isolation to provide an independent study group. This approach may redress the paucity of information on Andeans and fill gaps in our understanding of their evolutionary strategies for HA adaptation.

Our genome-wide analyses of population structure confirmed the genetic similarity between Colla, Quechua and Aymara groups. PCA and phylogenetic analyses based on genome-wide data grouped all three populations together. This tight clustering may either represent a signature of the early settlement of the Andes from the Pacific coast [95] or gene flow facilitated by the more recent expansion of the Inca Empire in the 15^th^ century across the Andean territory.

European admixture is low, both in Collas and Wichí, in contrast with the patterns of admixture observed in urban Argentinean populations [59]. This suggests that these groups have remained genetically isolated, despite the Spanish expansion during the conquest of the Americas in the 17^th^ century and the extensive post-war European immigration in the first half of the 20^th^ century. All mtDNA haplogroups clustered within Native American lineages, whereas 10–20% of Y-chromosome haplogroups were European, indicating moderate male biased gene flow. Analyses of the autosomal genome confirmed low levels of recent European admixture with genome-wide values of approximately 4% in Collas and 2% in Wichí (Table 2).

We carried out four different tests for positive selection aimed at detecting extended haplotype homozygosity (iHS and XP-EHH) and allele frequency differentiation (*F*_ST_ and PBS). The most prominent candidate gene identified by haplotype homozygosity tests in Collas is *VEGFB.* However, it is important to note that two other genes with a hypoxia-related function are also present in the same iHS window: *BAD*, encoding a hypoxia responsive protein involved in cell death regulation and *PRDX5*, a peroxisomal antioxidant enzyme that reduces hydrogen peroxide and is primarily expressed in mitochondria [96]. As iHS detects haplotypes that are both frequent in the population and longer than expected under the assumption of neutrality, it is hard to pinpoint the precise gene or variant that is driving the haplotype. The signal within the highest scoring window could thus be attributed to more than one gene, though *VEGFB* seems the most plausible candidate given its role in cardiac angiogenesis. This is also in line with the results from the XP-EHH test which highlighted *ELDT1*, another gene crucial for heart performance.

The angiogenic effect attributed to the VEGF-family is restricted for VEGF-β to the ischemic myocardium [97]. Insufficient blood supply and poor oxygenation in the heart have detrimental consequences at HA. Myocards relying on anaerobic metabolism accumulate lactate, which leads to water uptake by the cells and affects overall cellular function [98]. *VEGFB*-mediated angiogenesis, thus, may increase vascularisation of the myocardium and enhance cardiac output, ultimately improving oxygen supply to the whole body.

Genotype-phenotype correlations did not associate any phenotypic trait with the *VEGFB* haplotype; however, this result may be due to the small sample size, the traits considered or both. A bigger dataset may provide higher statistical power to detect an association, in particular if the putative causative mutation has a recessive effect and thus is only manifested in homozygote carriers. Moreover, the *VEGFB* haplotype could be associated with phenotypic traits not considered in this study. Phenotypic measurements were chosen to assess reported physiological Andean adaptations by non-invasive techniques. A direct measurement of haemoglobin concentration may add an important variable to future studies.

The estimated age of the *VEGFB* haplotype is approximately 3500 years, roughly coinciding with the emergence of the Quechua and Aymara languages [99]. Thus, the variant possibly arose shortly after the split of Quechua, Aymara and Collas, though it could have also arisen in the source population but has not yet been identified in the other two Andean groups. However, an important caveat to bear in mind is that the age estimates can be affected by sample size and by population history.

We investigated the possible functional implications of our findings by performing enrichment analysis of GO terms among the top 1% of iHS genes in Collas. Cardiac ventricle and chamber formation were among the enriched terms (Table S9). This, and the fact that *VEGFB* is predominantly expressed in the ischemic myocardium, suggests that the evolutionary advantage conferred by the putative selected variant of this gene may lie in its angiogenic role, endowing its carriers with a highly perfused and more efficient cardiac muscle, better equipped to provide adequate oxygen delivery in the presence of high blood viscosity. The selection of NO related GO terms further suggests vasodilation as an adaptive advantage by improving blood flow and oxygen distribution.

Another important candidate of selection is *ELTD1*, which was in the top two XP-EHH windows and was also identified by *F*_ST_ and PBS, albeit ranking 31^st^ and 145^th^ (among ca. 13,000 windows). This gene is thought to downregulate myocyte hypertrophy [89] as it is involved in the switch of cardiomyocytes from hyperplasia to hypertrophy [100]. It is thus possible that it was selected in Andeans to limit the extent of ventricular hypertrophy and prevent pathological effects such as those observed in CMS patients [101]. The Andean pulmonary artery was shown to be supported by an additional muscular layer to prevent damage from chronic pulmonary artery hypertension [26]. In addition to this adaptation, our findings suggest that selection on *ELTD1* and *VEGFB* may have resulted in further changes to the cardiovascular system to achieve efficient blood supply through controlled hypertrophy and increased perfusion of the myocard. Therefore, a complete suite of adaptations seems to have co-evolved, resulting in a reinforced and possibly more efficient cardiovascular system able to counteract the adverse effects of an increased haematocrit. In this regard it is interesting to note that our rough estimates indicate a significant reduction of cardiac output in highlanders (Table 3). Metabolic adaptations not yet identified coupled with increased oxygen carrying capacity may result in a decrease in oxygen demand at tissue level, beneficial at HA.

Besides *VEGFB* and *ELTD1*, *CBS* also appears to have been selected in Collas, ranking second in the PBS test. The gene was shown to increase cerebral blood flow (CBF) in mice during hypoxia [91]. CBF is reduced in Andeans [27] likely due to the elevated haematocrit and resulting blood viscosity. Thus, a possible role of *CBS* may be to counteract the decrease of CBF at HA and increase oxygen delivery to the brain.

We found little overlap between candidate genes identified by our study and those previously reported in HA populations. Five genes identified in the top 1% of the four selection tests in Collas have been previously suggested as candidates of positive selection (Table S12). Of these, only one (*PRKAA2*) was highlighted in Andeans [15]–[17]. *PRKAA2* is a protease inhibitor, important for energy balance in the ischemic heart [102]. If the top 5% of our four selection tests are considered, thirteen genes out of 5136 match previously highlighted genes in Andeans (Table S13). The small overlap may be due to the focus on a different, albeit related population, the different statistics employed, different tag SNPs used, our more stringent significance cut-off compared to previous studies [15], [16], or the lower power of detection of genotype data compared to genome sequence data [17]. Even though a similar combination of haplotype homozygosity (lnRH) and allele frequency differentiation tests (LSBL) was used by Bigham and colleagues, the exact ranking of genes could be different in the same data set if it was analysed with different statistics. *F*_ST_ and PBS were also employed by Zhou *et al* [17] but no overlapping candidate gene was detected with the same test. In this regard it is worth noting that Bigham *et al* [15] focused on a candidate gene list (75 HIF-pathway, 11 RAS and 27 globin genes) which only corresponded partially (30%) to our list of candidate genes. As Bigham *et al* [15], [16] reported results only for their candidate genes, many of the signatures discovered in our study may have a wider distribution among Andean highlanders. Interestingly, no overlap of candidate genes was found between Zhou *et al* [17] and Bigham *et al* [15], [16], even though both datasets included samples from the same region (Cerro de Pasco, Peru).

It is important to note that SNP ascertainment bias is inherent to whole genome scans [103], and since no Native American populations have been included in the ascertainment panels this bias may potentially affect the results of selection scans. At the same time, the design of tag-SNP chips has been shown to reduce the power to confirm those signals already detected in ascertainment panel (HapMap) populations and has a lesser effect on detecting new signals via haplotype homozygosity methods [74]. A more severe limitation of the genotype based selection tests is that genotyped SNPs represent only a subset of common variants rather than the likely true causative mutations. For an exhaustive list of selected genes whole genome sequences would be required. The selection tests employed in this study cover a variety of signatures of positive selection but are unable to detect soft sweeps acting on polygenic traits [104]. Another confounding factor may result from genetic drift. Villages in the Argentinean highlands are isolated, low heterozygosity has been described and a considerable amount of genetic drift suggested [44], [105]. Similarly, lowland Wichí communities often consist of a few families living together in one community. However, drift predominantly affects neutral variances equally across the genome, regardless of the gene's function [68], [106]. The finding of highly differentiated allele frequencies in Andeans Collas compared to Wichí together with strong signatures in genes with a putative role in the hypoxia response makes positive selection a more plausible force driving those particular signatures.

In summary, the analysis of genomic signatures of selection in Collas has enabled us to identify new mechanisms of adaptation, thus increasing understanding of the complexity and versatility of the hypoxic response. The most characteristic Andean adaptation to HA, namely the increased haematocrit, has a number of potentially adverse effects. To avoid these, a controlled reinforcement of the myocardium, improved cardiac perfusion, NO-mediated regulation of blood flow, control of oxidative damage and the offsetting of excessive CBF decrease seem to have developed to counteract maladaptation in the Collas. This array of adaptive strategies can be thought of as a bespoken evolutionary toolkit, distinct to that of Tibetans and Ethiopians, designed by nature to help Andean highlanders thrive in one of the most extreme environments on earth. Our work provides a new set of hypotheses which may open further avenues for research into conditions characterised by hypoxia and cardiac hypertrophy. Ultimately, this study advances our understanding of human adaptation and sheds light on the winding paths that nature takes to circumvent possible maladaptations.

## Supporting Information

Figure S1 **Sampling locations in the Province of Salta, Argentina.** Stars denote sampling locations; pink = highland locations of Collas, purple = lowland location of Wichí; Argentinean province names are displayed in italics. Altitudes of highland sampling locations: Tolar Grande (3524 m), Olacapato (4045 m), San Antonio de los Cobres (3775 m).
(DOCX)

Figure S2 **CV-errors for 100 runs from K = 2 to K = 10.** The lowest mean CV-error was observed at K = 6.
(DOCX)

Figure S3 **Log-likelihood difference between K = 2 to K = 10.** The lowest log likelihood difference (LL Diff) was observed at K = 2 to K = 4, while K = 6 and K = 8 also represent good fits for the data at higher K.
(DOCX)

Figure S4 **Representative admixture runs for K = 2 to K = 5.** At K = 2 the main admixture components are African (Yoruba) and Native Americans. K = 3 distinguishes HapMap Europeans sampled in the USA. K = 4 adds a component for Han Chinese and from K5 Wichí obtain their own admixture component. The sample origin is displayed above the admixture plot (Mex: Mexico, G: Guatemala, C: Colombia, B: Brazil, Arg: Argentina). Population name is listed underneath the plot (Kai/Cha/Gua/Tob: Kaingang from Brazil, Chané from Argentina, Guaraní from Argentina and Paraguay and Toba from Argentina; Chi/Cho/Hul/Yag: Chilote, Chono, Hulliche and Yaghan from Chile).
(TIF)

Figure S5 **One Mb region on chromosome 11 around** ***VEGFB*** **with genes of interest.** Crosses represent start and end points of genes, for details see Table S6. The highest scoring window of the iHS test in Collas was located at 64–64.2 Mb. As iHS is a haplotype test, surrounding areas may also influence the signal. Apart from the central region, the window upstream ranked 17^th^, windows downstream 11^th^ and 4^th^ among the top 1% of iHS windows.
(DOCX)

Table S1 **Y-chromosome haplogroup RFLP assay details.**
(DOCX)

Table S2 **Hypoxia candidate genes.**
(DOCX)

Table S3 **Comparison of mtDNA HV I molecular diversity estimates among Amerindians** [**1**] **.**
(DOCX)

Table S4 **mtDNA haplotypes and haplogroup assignment of Collas and Wichí.**
(DOCX)

Table S5 **Top 1% of iHS and XP-EHH results in Collas.**
(XLSX)

Table S6 **Genes of interest in the 1 Mb region around** ***VEGFB*** **.**
(DOCX)

Table S7 **Candidate genes in the top 1% of PBS results in Collas.**
(DOCX)

Table S8 **Genes detected with more than one selection test in Collas.**
(DOCX)

Table S9 **GO terms enrichment of the top 1% iHS windows in Collas with EASE-score <0.01.**
(DOCX)

Table S10 **Enriched GO terms in the XP-EHH top 1% in Collas.**
(DOCX)

Table S11 **GO term enrichment of PBS genes in the top 1% in Collas.**
(DOCX)

Table S12 **Hypoxia genes identified in this study and in other HA studies.**
(DOCX)

Table S13 **Gene overlap in the top 5% of this study and Bigham** ***et al*** **[1],[2]** **and Zhou** ***et al*** **[3]** **.**
(DOCX)
