# Supplementary material for: The Andean Adaptive Toolkit to Counteract High Altitude Maladaptation: Genome-Wide and Phenotypic Analysis of the Collas
Source: PLoS One. 2014 Mar 31;9(3):e93314. doi: 10.1371/journal.pone.0093314 (PMC3970967; doi:10.1371/journal.pone.0093314)
Supplement: Figure S1 — Sampling locations in the Province of Salta, Argentina. Stars denote sampling locations; pink = highland locations of Collas, purple = lowland location of Wichí; Argentinean province names are displayed in italics. Altitudes of highland sampling locations: Tolar Grande (3524 m), Olacapato (4045 m), San Antonio de los Cobres (3775 m). (DOCX) [file pone.0093314.s001.docx]

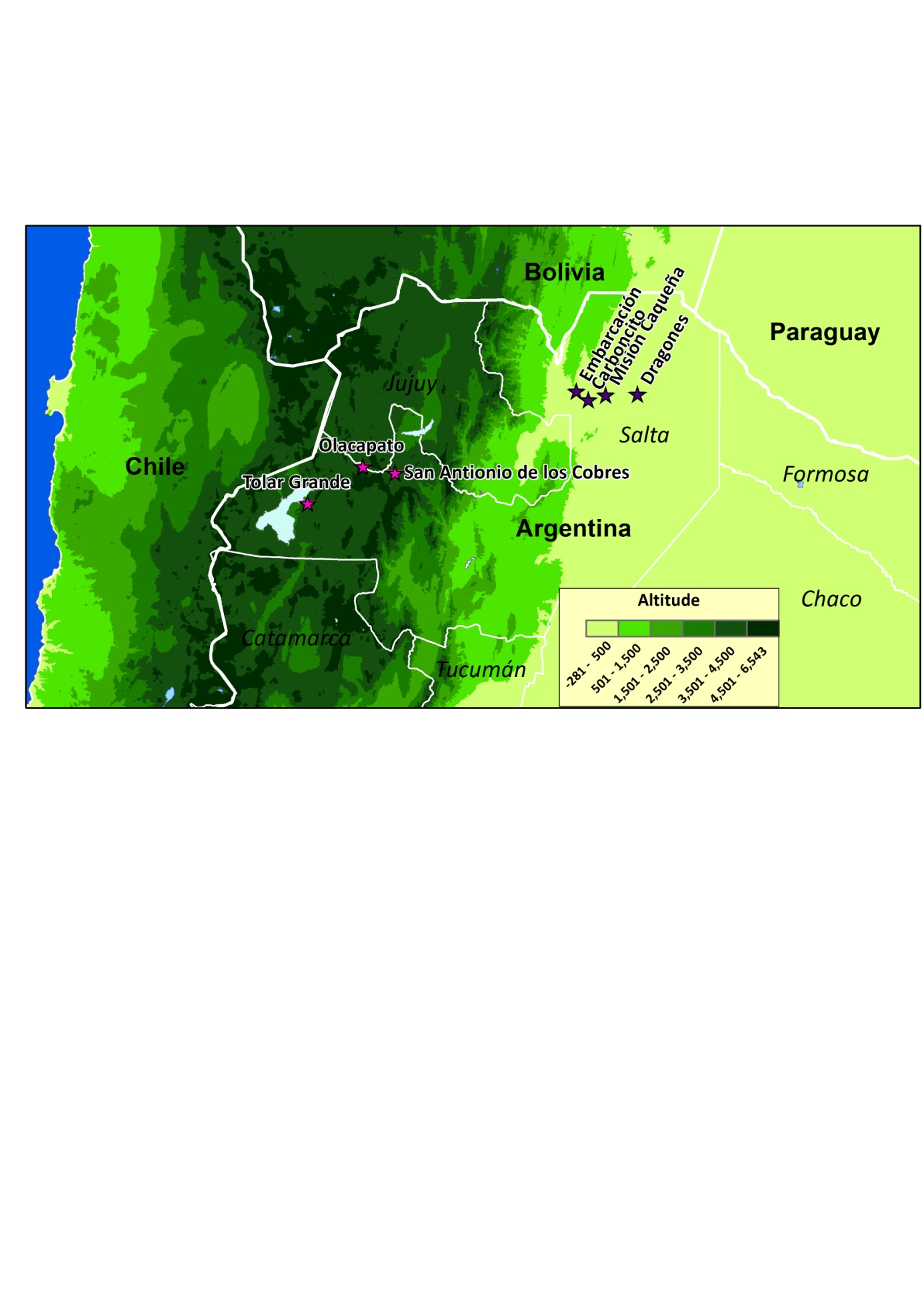


Figure S1. Sampling locations in the Province of Salta, Argentina.

Stars denote sampling locations; pink= highland locations of Colla, purple= lowland location of Wichí; Argentinean province names are displayed in italics. Altitudes of highland sampling locations: Tolar Grande (3524 m), Olacapato (4045 m), San Antonio de los Cobres (3775 m).
