# Supplementary material for: The Andean Adaptive Toolkit to Counteract High Altitude Maladaptation: Genome-Wide and Phenotypic Analysis of the Collas
Source: PLoS One. 2014 Mar 31;9(3):e93314. doi: 10.1371/journal.pone.0093314 (PMC3970967; doi:10.1371/journal.pone.0093314)
Supplement: Figure S2 — CV-errors for 100 runs from K = 2 to K = 10. The lowest mean CV-error was observed at K = 6. (DOCX) [file pone.0093314.s002.docx]

Figure S2. CV-errors for 100 runs from K=2 to K=10.
The lowest mean CV-error was observed at K=6.
