# Supplementary material for: The Andean Adaptive Toolkit to Counteract High Altitude Maladaptation: Genome-Wide and Phenotypic Analysis of the Collas
Source: PLoS One. 2014 Mar 31;9(3):e93314. doi: 10.1371/journal.pone.0093314 (PMC3970967; doi:10.1371/journal.pone.0093314)
Supplement: Figure S3 — Log-likelihood difference between K = 2 to K = 10. The lowest log likelihood difference (LL Diff) was observed at K = 2 to K = 4, while K = 6 and K = 8 also represent good fits for the data at higher K. (DOCX) [file pone.0093314.s003.docx]

Figure S3. Log-likelihood difference between K=2 to K=10.
The lowest log likelihood difference (LL Diff) was observed at K=2 to K=4, while K=6 and K=8 also represent good fits for the data at higher K.
