# Supplementary figures and images for: The Andean Adaptive Toolkit to Counteract High Altitude Maladaptation: Genome-Wide and Phenotypic Analysis of the Collas
Source: PLoS One. 2014 Mar 31;9(3):e93314. doi: 10.1371/journal.pone.0093314 (PMC3970967; doi:10.1371/journal.pone.0093314)

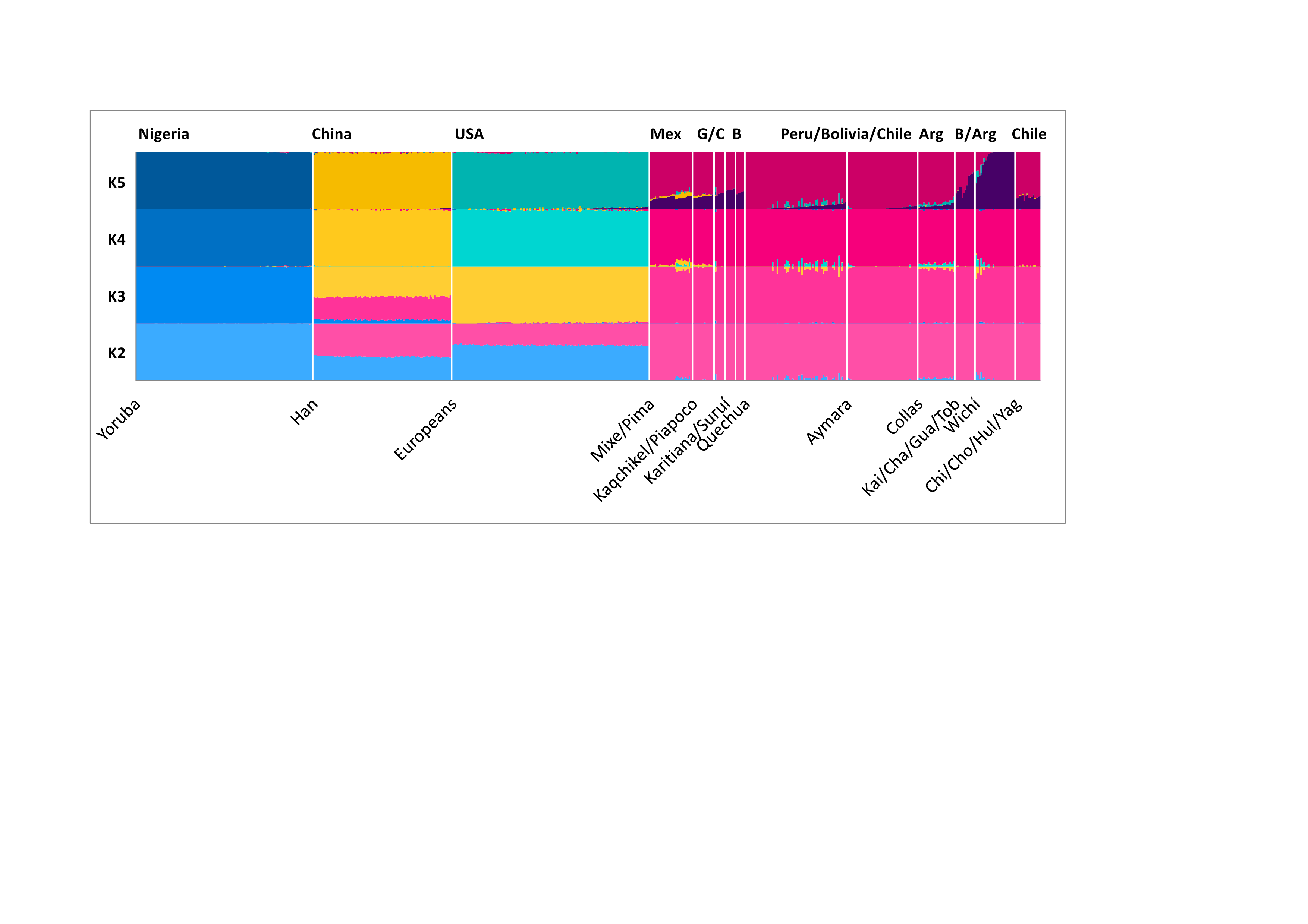

Supplement: Figure S4 — Representative admixture runs for K = 2 to K = 5. At K = 2 the main admixture components are African (Yoruba) and Native Americans. K = 3 distinguishes HapMap Europeans sampled in the USA. K = 4 adds a component for Han Chinese and from K5 Wichí obtain their own admixture component. The sample origin is displayed above the admixture plot (Mex: Mexico, G: Guatemala, C: Colombia, B: Brazil, Arg: Argentina). Population name is listed underneath the plot (Kai/Cha/Gua/Tob: Kaingang from Brazil, Chané from Argentina, Guaraní from Argentina and Paraguay and Toba from Argentina; Chi/Cho/Hul/Yag: Chilote, Chono, Hulliche and Yaghan from Chile). (TIF) [file pone.0093314.s004.tif]
