# Supplementary material for: The Andean Adaptive Toolkit to Counteract High Altitude Maladaptation: Genome-Wide and Phenotypic Analysis of the Collas
Source: PLoS One. 2014 Mar 31;9(3):e93314. doi: 10.1371/journal.pone.0093314 (PMC3970967; doi:10.1371/journal.pone.0093314)
Supplement: Figure S5 — One Mb region on chromosome 11 around VEGFB with genes of interest. Crosses represent start and end points of genes, for details see Table S6. The highest scoring window of the iHS test in Collas was located at 64–64.2 Mb. As iHS is a haplotype test, surrounding areas may also influence the signal. Apart from the central region, the window upstream ranked 17th, windows downstream 11th and 4th among the top 1% of iHS windows. (DOCX) [file pone.0093314.s005.docx]

**Window Rank:** *#17 #1 #11 #4*

Figure S5. One Mb region on chromosome 11 around *VEGFB* with genes of interest.

Crosses represent start and end points of genes, for details see Table S3. The highest scoring window of the iHS test in Collas was located at 64-64.2 Mb. As iHS is a haplotype test, surrounding areas may also influence the signal. Apart from the central region, the window upstream ranked 17^th^, windows downstream 11^th^ and 4^th^ among the top 1% of iHS windows.
