# Supplementary material for: The Andean Adaptive Toolkit to Counteract High Altitude Maladaptation: Genome-Wide and Phenotypic Analysis of the Collas
Source: PLoS One. 2014 Mar 31;9(3):e93314. doi: 10.1371/journal.pone.0093314 (PMC3970967; doi:10.1371/journal.pone.0093314)
Supplement: Table S1 — Y-chromosome haplogroup RFLP assay details. (DOCX) [file pone.0093314.s006.docx]

Table S1. Y-chromosome haplogroup RFLP assay details.

| **Haplogroup** | **Enzyme site** | **Primer 5’-3’** | **Restriction pattern** |
| --- | --- | --- | --- |
| Q | Alw21 I | Fwd: AACTCTTGATAAACCGTGCTG | C: 179+187 |
|  | gwgcw/**c** | Rev: TCCAATCTCAATTCATGCCTC | T: 366 |
| R1b | BstN I | Fwd: CTAAAGATCAGAGTATCTCCCTTTG | C: 303+125 |
|  | **c**c/wgg | Rev: ACTATACTTCTTTTGTGTGCCTTC [[1](#_ENREF_1)] | T: 428 |

**Supplemental Reference**

1. Cruciani F, Santolamazza P, Shen P, Macaulay V, Moral P, et al. (2002) A back migration from Asia to sub-Saharan Africa is supported by high-resolution analysis of human Y-chromosome haplotypes. Am J Hum Genet 70: 1197-1214.
