# Supplementary material for: The Andean Adaptive Toolkit to Counteract High Altitude Maladaptation: Genome-Wide and Phenotypic Analysis of the Collas
Source: PLoS One. 2014 Mar 31;9(3):e93314. doi: 10.1371/journal.pone.0093314 (PMC3970967; doi:10.1371/journal.pone.0093314)
Supplement: Table S2 — Hypoxia candidate genes. (DOCX) [file pone.0093314.s007.docx]

Table S2. Hypoxia candidate genes.

| **Gene** | **Name** | **Pathway** |
| --- | --- | --- |
| *ADAM8* | Disintegrin and metalloproteinase domain-containing protein 8 | **GO term: ‘Cellular response to hypoxia’** |
| *AJUBA* | LIM domain-containing protein ajuba |  |
| *AKT1* | RAC-alpha serine/threonine-protein kinase |  |
| *ANGPT4* | Angiopoietin-4 |  |
| *ANKRD1* | Ankyrin repeat domain-containing protein 1 |  |
| *AQP1* | Aquaporin-1 |  |
| *ARNT* | Aryl hydrocarbon receptor nuclear translocator |  |
| *BACH1* | Transcription regulator protein BACH1 |  |
| *BAD* | Bcl2 antagonist of cell death |  |
| *BBC3* | Bcl-2-binding component 3 |  |
| *BMP7* | Bone morphogenetic protein 7 |  |
| *BNIP3* | BCL2/adenovirus E1B 19 kDa protein-interacting protein 3 |  |
| *CA9* | Carbonic anhydrase 9 |  |
| *CBS* | Cysteine synthase |  |
| *CCNB1* | G2/mitotic-specific cyclin-B1 |  |
| *CD34* | Haematopoietic progenitor cell antigen CD34 |  |
| *CITED2* | Cbp/p300-interacting transactivator 2 |  |
| *CREBBP* | CREB-binding protein |  |
| *CUL2* | Cullin-2 |  |
| *E2F1* | Transcription factor E2F1 |  |
| *EGLN1* | Egl nine homolog 1 |  |
| *EGLN2* | Egl nine homolog 2 |  |
| *EGLN3* | Egl nine homolog 3 |  |
| *EGR1* | Early growth response protein 1 |  |
| *EP300* | Histone acetyltransferase p300 |  |
| *EPAS1* | Endothelial PAS domain-containing protein 1 |  |
| *EPO* | Erythropoietin |  |
| *FABP1* | Fatty acid-binding protein, liver |  |
| *FAM162A* | Protein FAM162A |  |
| *FMN2* | Formin-2 |  |
| *FNDC1* | Fibronectin type III domain-containing protein 1 |  |
| *GATA6* | Transcription factor GATA-6 |  |
| *GNB1* | Guanine nucleotide-binding protein G(I)/G(S)/G(T) subunit β-1 |  |
| *GNGT1* | Guanine nucleotide-binding protein G(T) subunit gamma-T1 |  |
| *HIF1A* | Hypoxia-inducible factor 1-alpha |  |
| *HIF1AN* | Hypoxia-inducible factor 1-alpha inhibitor |  |
| *HIF3A* | Hypoxia-inducible factor 3-alpha |  |
| *HIPK2* | Homeodomain-interacting protein kinase 2 |  |
| *HMOX1* | Haem oxygenase 1 |  |
| *ICAM1* | Intercellular adhesion molecule 1 |  |
| *IRAK1* | Interleukin-1 receptor-associated kinase 1 |  |
| *IREB2* | Iron-responsive element-binding protein 2 |  |
| *KCNK3* | Potassium channel subfamily K member 3 |  |
| *LMNA* | Prelamin-A/C |  |
| *MDM2* | E3 ubiquitin-protein ligase Mdm2 |  |
| *MDM4* | Protein Mdm4 |  |
| *MGARP* | Protein MGARP |  |
| *MT3* | Metallothionein-3 |  |
| *MTOR* | Serine/threonine-protein kinase mTOR |  |
| *MYOCD* | Myocardin |  |
| *NDRG1* | Protein NDRG1 |  |
| *NKX3-1* | Homeobox protein Nkx-3.1 |  |
| *NOTCH1* | Neurogenic locus notch homolog protein 1 |  |
| *NPEPPS* | Puromycin-sensitive aminopeptidase |  |
| *PDIA2* | Protein disulfide-isomerase A2 | **GO term: ‘Cellular response to hypoxia’** |
| *PDK1* | Pyruvate dehydrogenase kinase isozyme 1, mitochondrial |  |
| *PDK3* | Pyruvate dehydrogenase kinase isozyme 3, mitochondrial |  |
| *PLK3* | Serine/threonine-protein kinase PLK3 |  |
| *PMAIP1* | Phorbol-12-myristate-13-acetate-induced protein 1 |  |
| *PRKAA1* | 5&#39;-AMP-activated protein kinase catalytic subunit alpha-1 |  |
| *PRKCE* | Protein kinase C epsilon type |  |
| *PTGIS* | Prostacyclin synthase |  |
| *PTGS2* | Prostaglandin G/H synthase 2 |  |
| *RBX1* | E3 ubiquitin-protein ligase RBX1 |  |
| *RGCC* | Regulator of cell cycle RGCC |  |
| *RPS27A* | Ubiquitin-40S ribosomal protein S27a |  |
| *S100B* | Protein S100-B |  |
| *SFRP1* | Secreted frizzled-related protein 1 |  |
| *SIRT1* | NAD-dependent protein deacetylase sirtuin-1 |  |
| *SLC29A1* | Equilibrative nucleoside transporter 1 |  |
| *STC1* | Stanniocalcin-1 |  |
| *STC2* | Stanniocalcin-2 |  |
| *TCEB1* | Transcription elongation factor B polypeptide 1 |  |
| *TCEB2* | Transcription elongation factor B polypeptide 2 |  |
| *TP53* | Cellular tumour antigen p53 |  |
| *TWIST1* | Twist-related protein 1 |  |
| *UBA52* | Ubiquitin-60S ribosomal protein L40 |  |
| *UBB* | Polyubiquitin-B |  |
| *UBC* | Polyubiquitin-C |  |
| *UBE2D1* | Ubiquitin-conjugating enzyme E2 D1 |  |
| *UBE2D2* | Ubiquitin-conjugating enzyme E2 D2 |  |
| *UBE2D3* | Ubiquitin-conjugating enzyme E2 D3 |  |
| *UBQLN1* | Ubiquilin-1 |  |
| *UCN2* | Urocortin-2 |  |
| *UCN3* | Urocortin-3 |  |
| *USP19* | Ubiquitin carboxyl-terminal hydrolase 19 |  |
| *VEGFA* | Vascular endothelial growth factor A |  |
| *VHL* | Von Hippel-Lindau disease tumour suppressor |  |
| *GUCY1A2* | Guanylate cyclase soluble subunit alpha-2 [cytosol] | **REACTOME pathway: ‘NO stimulates guanylate cyclase’** |
| *GUCY1A3* | Guanylate cyclase soluble subunit alpha-3 [cytosol] |  |
| *GUCY1B2* | Guanylate cyclase soluble subunit β-2 [cytosol] |  |
| *GUCY1B3* | Guanylate cyclase soluble subunit β-1 [cytosol] |  |
| *ITPR1* | IP3 receptor type 1 [platelet dense tubular network membrane] |  |
| *KCNMA1* | Calcium-activated potassium channel subunit α-1 [plasma membrane] |  |
| *KCNMB1* | Calcium-activated potassium channel subunit β-1 [plasma membrane] |  |
| *KCNMB2* | Calcium-activated potassium channel subunit β-2 [plasma membrane] |  |
| *KCNMB3* | Calcium-activated potassium channel subunit β-3 [plasma membrane] |  |
| *KCNMB4* | Calcium-activated potassium channel subunit β-4 [plasma membrane] |  |
| *MRVI1* | IRAG [platelet dense tubular network membrane] |  |
| *NOS1* | NOS1 [cytosol] |  |
| *NOS2* | Nitric oxide synthase, inducible [cytosol] |  |
| *NOS3* | Nitric oxide synthase, endothelial [cytosol] |  |
| *PDE10A* | Phosphodiesterase 10A [cytosol] |  |
| *PDE11A* | Phosphodiesterase 11A [cytosol] |  |
| *PDE1A* | Phosphodiesterase 1A, calcium/calmodulin-dependent [cytosol] |  |
| *PDE1B* | Phosphodiesterase 1B, calcium/calmodulin-dependent [cytosol] |  |
| *PDE2A* | Phosphodiesterase 2A [cytosol] |  |
| *PDE3A* | Phosphodiesterase 3A [cytosol] |  |
| *PDE3B* | Phosphodiesterase 3B [cytosol] |  |
| *PDE5A* | Phosphodiesterase 5A [cytosol] |  |
| *PDE6A* | PDE6A [photoreceptor disc membrane] |  |
| *PDE6B* | PDE6B [photoreceptor disc membrane] | **REACTOME pathway: ‘NO stimulates guanylate cyclase’** |
| *PDE6G* | PDE6G [photoreceptor disc membrane] |  |
| *PDE9A* | Phosphodiesterase 9A [cytosol] |  |
| *PRKG1* | cGMP-dependent protein kinase 1, alpha isozyme [cytosol] |  |
| *PRKG2* | PRKG2 [cytosol] |  |
| *AGAP3* | Arf-GAP with GTPase, ANK repeat and PH domain-containing protein 3 | **GO term: ‘Cellular response to ROS’** |
| *AKR1C3* | Aldo-keto reductase family 1 member C3 |  |
| *ANXA1* | Annexin A1 |  |
| *APEX1* | DNA-(apurinic or apyrimidinic site) lyase |  |
| *APOA4* | Apolipoprotein A-IV |  |
| *ARG1* | Arginase-1 |  |
| *ATP7A* | Copper-transporting ATPase 1 |  |
| *CAT* | Catalase |  |
| *CCS* | Copper chaperone for superoxide dismutase |  |
| *CD36* | Platelet glycoprotein 4 |  |
| *CDK1* | Cyclin-dependent kinase 1 |  |
| *CDK2* | Cyclin-dependent kinase 2 |  |
| *CRYGD* | Gamma-crystallin D |  |
| *CST3* | Cystatin-C |  |
| *DPEP1* | Dipeptidase 1 |  |
| *DUOX1* | Dual oxidase 1 |  |
| *DUOX2* | Dual oxidase 2 |  |
| *ECT2* | Protein ECT2 |  |
| *EPX* | Eosinophil peroxidase |  |
| *ETS1* | Protein C-ets-1 |  |
| *FANCC* | Fanconi anaemia group C protein |  |
| *FBLN5* | Fibulin-5 |  |
| *FER* | Tyrosine-protein kinase Fer |  |
| *FOS* | Proto-oncogene c-Fos |  |
| *FOXO1* | Forkhead box protein O1 |  |
| *FXN* | Frataxin, mitochondrial |  |
| *GPX1* | Glutathione peroxidase 1 |  |
| *GPX3* | Glutathione peroxidase 3 |  |
| *HBA1* | Haemoglobin subunit alpha |  |
| *HBB* | Haemoglobin subunit β |  |
| *HDAC6* | Histone deacetylase 6 |  |
| *HGF* | Hepatocyte growth factor |  |
| *HP* | Haptoglobin |  |
| *IL18* | Interleukin-18 |  |
| *IL18BP* | Interleukin-18-binding protein |  |
| *IL6* | Interleukin-6 |  |
| *KLF2* | Krueppel-like factor 2 |  |
| *LCN2* | Neutrophil gelatinase-associated lipocalin |  |
| *LPO* | Lactoperoxidase |  |
| *MAP3K5* | Mitogen-activated protein kinase kinase kinase 5 |  |
| *MAPK7* | Mitogen-activated protein kinase 7 |  |
| *MET* | Hepatocyte growth factor receptor |  |
| *MPO* | Myeloperoxidase |  |
| *MPV17* | Protein Mpv17 |  |
| *NET1* | Neuroepithelial cell-transforming gene 1 protein |  |
| *NFE2L2* | Nuclear factor erythroid 2-related factor 2 |  |
| *PARK7* | Protein DJ-1 |  |
| *PAX2* | Paired box protein Pax-2 |  |
| *PDK2* | [Pyruvate dehydrogenase [lipoamide]] kinase isozyme 2, mitochondrial |  |
| *PLEKHA1* | Pleckstrin homology domain-containing family A member 1 |  |
| *PPARGC1B* | Peroxisome proliferator-activated receptor gamma coactivator 1-β |  |
| *PPIF* | Peptidyl-prolyl cis-trans isomerase F, mitochondrial | **GO term: ‘Cellular response to ROS’** |
| *PRDX1* | Peroxiredoxin-1 |  |
| *PRDX2* | Peroxiredoxin-2 |  |
| *PRDX3* | Thioredoxin-dependent peroxide reductase, mitochondrial |  |
| *PRDX5* | Peroxiredoxin-5, mitochondrial |  |
| *PRDX6* | Peroxiredoxin-6 |  |
| *PTPRK* | Receptor-type tyrosine-protein phosphatase kappa |  |
| *PXDN* | Peroxidasin homolog |  |
| *PXDNL* | Peroxidasin-like protein |  |
| *PXN* | Paxillin |  |
| *RHOB* | Rho-related GTP-binding protein RhoB |  |
| *ROMO1* | Reactive oxygen species modulator 1 |  |
| *SFTPC* | Pulmonary surfactant-associated protein C |  |
| *SLC8A1* | Sodium/calcium exchanger 1 |  |
| *SOD1* | Superoxide dismutase [Cu-Zn] |  |
| *SOD2* | Superoxide dismutase |  |
| *SOD3* | Extracellular superoxide dismutase [Cu-Zn] |  |
| *TNFAIP3* | Tumour necrosis factor alpha-induced protein 3 |  |
| *TPM1* | Tropomyosin alpha-1 chain |  |
| *TPO* | Thyroid peroxidase |  |
| *TXNRD1* | Thioredoxin reductase 1, cytoplasmic |  |
| *ACE* | ACE [plasma membrane] | **REACTOME pathway: ‘Angiotensinogen to angiotensins’** |
| *ACE2* | ACE2 [plasma membrane] |  |
| *AGT* | Angiotensin-(1-10) [extracellular region] |  |
| *ANPEP* | ANPEP [plasma membrane] |  |
| *ATP6AP2* | Prorenin receptor [plasma membrane] |  |
| *CMA1* | Chymase [extracellular region] |  |
| *CPA3* | CPA3 [extracellular region] |  |
| *CTSD* | CTSD [extracellular region] |  |
| *CTSG* | Cathepsin G [plasma membrane] |  |
| *CTSZ* | Cathepsin Z [plasma membrane] |  |
| *ENPEP* | ENPEP [plasma membrane] |  |
| *GZMH* | GZMH [plasma membrane] |  |
| *MME* | Neprilysin [plasma membrane] |  |
| *REN* | Prorenin [plasma membrane] |  |
| *FIGF* | VEGFD [extracellular region] | **REACTOME pathway: ‘VEGF signalling’** |
| *FLT1* | VEGFR1 [plasma membrane] |  |
| *FLT4* | VEGFR3 [plasma membrane] |  |
| *KDR* | VEGFR2 [plasma membrane] |  |
| *NRP1* | NRP1 [plasma membrane] |  |
| *NRP2* | NRP2 [plasma membrane] |  |
| *PDGFC* | VEGFE [extracellular region] |  |
| *PGF* | PGF [extracellular region] |  |
| *VEGFB* | VEGFB [extracellular region] |  |
| *VEGFC* | VEGFC [extracellular region] |  |
