# Supplementary material for: The Andean Adaptive Toolkit to Counteract High Altitude Maladaptation: Genome-Wide and Phenotypic Analysis of the Collas
Source: PLoS One. 2014 Mar 31;9(3):e93314. doi: 10.1371/journal.pone.0093314 (PMC3970967; doi:10.1371/journal.pone.0093314)
Supplement: Table S3 — Comparison of mtDNA HV I molecular diversity estimates among Amerindians [1] . (DOCX) [file pone.0093314.s008.docx]

Table S3. Comparison of mtDNA HV I molecular diversity estimates among Amerindians [[1](#_ENREF_1)].

| **Population** | ***h*** | ***θ_π_* (SD)** | **Original study** |
| --- | --- | --- | --- |
| *Chibchans* |  |  |  |
| Ngobe | 0.763 | 5.198 (2.844) | [[2](#_ENREF_2)] |
| Arsario | 0.725 | 4.878 (2.729) | [[3](#_ENREF_3)] |
| Huetar | 0.709 | 4.018 (2.307) | [[4](#_ENREF_4)] |
| Kuna | 0.592 | 3.882 (2.190) | [[5](#_ENREF_5)] |
| Kogi | 0.524 | 3.851 (2.249) | [[3](#_ENREF_3)] |
| Ijka | 0.184 | 1.728 (1.151) | [[3](#_ENREF_3)] |
|  |  |  |  |
| *Western Lowland South Americans* |  |  |  |
| Embera | 0.940 | 6.673 (3.563) | [[6](#_ENREF_6)] |
| Wounan | 0.912 | 7.569 (4.039) | [[6](#_ENREF_6)] |
| Cayapa | 0.837 | 7.253 (3.888) | [[7](#_ENREF_7)] |
|  |  |  |  |
| *North Central Andes* |  |  |  |
| Ancash | 0.981 | 6.869 (3.688) | [[8](#_ENREF_8)] |
| Tayacaja | 0.967 | 7.087 (3.739) | [[9](#_ENREF_9)] |
| Yungay | 0.954 | 6.203 (3.353) | [[10](#_ENREF_10)] |
| Tupe | 0.867 | 6.426 (3.598) | [[10](#_ENREF_10)] |
|  |  |  |  |
| *South Central Andes* |  |  |  |
| Arequipa (Quechua) | 0.978 | 5.961 (3.298) | [[9](#_ENREF_9)] |
| Aymara Chile | 0.973 | 5.843 (3.170) | [[11](#_ENREF_11)] |
| Quechua Puno 1 | 0.972 | 6.150 (3.348) | [[10](#_ENREF_10)] |
| **Collas** | **0.957** | **5.973 (3.284)** | **This study** |
| Coya | 0.970 | 6.868 (3.635) | [[12](#_ENREF_12)] |
| Aymara Puno 1 | 0.967 | 5.701 (3.263) | [[10](#_ENREF_10)] |
| Aymara Lake Titicaca | 0.960 | 4.904 (2.670) | [[13](#_ENREF_13)] |
| Quechua Puno 2 | 0.960 | 6.659 (3.573) | [[14](#_ENREF_14)] |
| Aymara La Paz | 0.950 | 5.843 (3.135) | [[1](#_ENREF_1)] |
| Aymara Puno 2 | 0.947 | 3.760 (2.210) | [[14](#_ENREF_14)] |
| Atacameno | 0.947 | 7.365 (3.954) | [[11](#_ENREF_11)] |
| Quechua Potosi | 0.939 | 6.447 (3.411) | [[13](#_ENREF_13)] |
|  |  |  |  |
| *Southern Andes* |  |  |  |
| Huilliche | 0.935 | 6.838 (3.623) | [[11](#_ENREF_11)] |
| Tehuelche | 0.916 | 6.394 (3.472) | [[11](#_ENREF_11)] |
| Mapuche (Argentina) | 0.908 | 6.427 (3.455) | [[15](#_ENREF_15)] |
| Yaghan | 0.886 | 6.436 (3.635) | [[16](#_ENREF_16)] |
| Mapuche/Pehuenche | 0.875 | 6.823 (3.616) | [[16](#_ENREF_16)] |
|  |  |  |  |
| *Lowland Bolivians, Department of Beni* |  |  |  |
| Moxo | 0.960 | 7.329 (3.942) | [[17](#_ENREF_17)] |
| Yuracare | 0.943 | 7.326 (4.073) | [[17](#_ENREF_17)] |
| Chimane/Moseten | 0.926 | 6.599 (3.634) | [[18](#_ENREF_18)] |
| Movima | 0.894 | 3.319 (2.063) | [[17](#_ENREF_17)] |
| Quechua Beni | 0.758 | 5.163 (2.956) | [[18](#_ENREF_18)] |
|  |  |  |  |
| *Gran Chaco* |  |  |  |
| Pilaga | 0.963 | 7.843 (4.147) | [[19](#_ENREF_19)] |
| **Wichí** | **0.900** | **7.207 (3.580)** | **This study** |
| Wichi | 0.896 | 6.798 (3.573) | [[19](#_ENREF_19)] |
| Toba | 0.869 | 5.848 (3.138) | [[19](#_ENREF_19)] |
|  |  |  |  |
| *Other lowland South Americans* |  |  |  |
| Yanomamo | 0.906 | 5.554 (2.974) | [[20](#_ENREF_20)] |
| Guahibo | 0.858 | 5.800 (3.121) | [[21](#_ENREF_21)] |
| Nandeva | 0.844 | 3.876 (2.192) | [[22](#_ENREF_22)] |
| Zoro/Gaviao | 0.842 | 4.916 (2.695) | [[23](#_ENREF_23)] |
| Wayuu | 0.773 | 6.739 (3.641) | [[22](#_ENREF_22)] |
| Kaingang | 0.744 | 6.883 (3.630) | [[22](#_ENREF_22)] |
| Xavante | 0.677 | 3.474 (2.043) | [[23](#_ENREF_23)] |
| M'bya | 0.652 | 2.707 (1.661) | [[22](#_ENREF_22)] |
| Kaiowa | 0.593 | 1.791 (1.154) | [[22](#_ENREF_22)] |
| Ayoreo | 0.473 | 2.761 (1.635) | [[24](#_ENREF_24)] |
| Ache | 0.204 | 1.241 (0.884) | [[25](#_ENREF_25)] |

**Supplemental References**

1. Batai K, Williams SR (2014) Mitochondrial variation among the aymara and the signatures of population expansion in the central Andes. Am J Hum Biol.

2. Kolman CJ, Bermingham E, Cooke R, ward RH, Arias TD, et al. (1995) Reduced mtDNA diversity in the Ngöbé Amerinds of Panamá. Genetics 140: 275-283.

3. Melton PE, Briceño I, Gómez A, Devor EJ, Bernal JE, et al. (2007) Biological relationship between Central and South American Chibchan speaking populations: evidence from mtDNA. American Journal of Physical Anthropology 133: 753-770.

4. Santos M, Ward RH, Barrantes R (1994) mtDNA variation in the Chibcha Amerindian Huetar from Costa Rica. Human Biology 66: 963-977.

5. Batista O, Kolman CJ, Bermingham E (1995) Mitochondrial DNA diversity in the Kuna Amerinds of Panamá. Human Molecular Genetics 4: 921-929.

6. Kolman CJ, Bermingham E (1997) Mitochondrial and nuclear DNA diversity in the Choco and Chibcha Amerinds of Panama. Genetics 147: 1289-1302.

7. Rickards O, Martínez-Labarga C, Lum JK, De Stefano GF, Cann RL (1999) mtDNA history of the Cayapa Amerinds of Ecuador: detection of additional founding lineages for the Native American populations. American Journal of Human Genetics 65: 519-530.

8. Lewis CMJ, Tito RY, Lizarraga B, Stone AC (2005) Land, language, and loci: mtDNA in Native Americans and the genetic history of Peru. Am J Phys Anthropol 127: 351-360.

9. Fuselli S, Tarazona-Santos E, Dupanloup I, Soto A, Luiselli D, et al. (2003) Mitochondrial DNA diversity in South America and the genetic history of Andean highlanders. Mol Biol Evol 20: 1682-1691.

10. Lewis CMJ, Lizárraga B, Tito RY, López PW, Iannacone GC, et al. (2007) Mitochondrial DNA and the peopling of South America. Hum Biol 79: 159-178.

11. de Saint Pierre M, Bravi CM, Motti JMB, Fuku N, Tanaka M, et al. (2012) An Alternative Model for the Early Peopling of Southern South America Revealed by Analyses of Three Mitochondrial DNA Haplogroups. PLoS ONE 7: e43486.

12. Álvarez-Iglesias V, Jaime JC, Carracedo Á, Salas A (2007) Coding region mitochondrial DNA SNPs: Targeting East Asian and Native American haplogroups. Forensic Sci Int Genet 1: 44-55.

13. Gayà-Vidal M, Moral P, Saenz-Ruales N, Gerbault P, Tonasso L, et al. (2011) mtDNA and Y-chromosome diversity in Aymaras and Quechuas from Bolivia: Different stories and special genetic traits of the Andean Altiplano populations. Am J Phys Anthropol 145: 215-230.

14. Barbieri C, Heggarty P, Castrì L, Luiselli D, Pettener D (2011) Mitochondrial DNA variability in the Titicaca basin: Matches and mismatches with linguistics and ethnohistory. Am J Hum Biol 23: 89-99.

15. Ginther C, Corach D, Penacino A, Rey JA, Carnese FR, et al. (1993) Genetic variation among the Mapuche Indians from the Patagonian region of Argentina: Mitochondrial DNA sequence variation and allele frequencies of several nuclear genes. In: Penn SDJ, Chakraborty R, Epplen JT, Jeffreys A, editors. DNA Fingerprinting: State of the Science. Basel, Switzerland: Birkhäuser Verlag. pp. 211-219.

16. Moraga ML, Rocco P, Miquel JF, Nervi F, Llop E, et al. (2000) Mitochondrial DNA polymorphisms in Chilean aboriginal populations: implications for the peopling of the southern cone of the continent. Am J Phys Anthropol 113: 19-29.

17. Bert F, Corella A, Gene M, Perez-Perez A, Turbon D (2004) Mitochondrial DNA diversity in the Llanos de Moxos: Moxo, Movima and Yuracare Amerindian populations from Bolivia lowland. Ann Hum Biol 31: 9-28.

18. Corella A, Bert F, Pérez-Pérez A, Gen M, Turbón D (2007) Mitochondrial DNA diversity of the Amerindian populations living in the Andean Piedmont of Bolivia: Chimane, Moseten, Aymara and Quechua. Ann Hum Biol 34: 34-55.

19. Cabana GS, Merriwether DA, Hunley K, Demarchi DA (2006) Is the genetic structure of Gran Chaco populations unique? Interregional perspectives on native South American mitochondrial DNA variation. Am J Phys Anthropol 131: 108-119.

20. Merriwether DA, Kemp BM, Crews DE, Neel JV (2000) Gene flow and genetic variation in the Yanomama as revealed by mitochondrial DNA. In: Renfrew C, editor. America Past, America Present, Genes and Languages in the Americas and Beyond. Cambridge: The McDonald Institute for Archaeological Research. pp. 84-124.

21. Vona G, Falchi A, Moral P, Caló C, Varesi L (2006) Mitochondrial sequence variation in the Guahibo Amerindian population from Venezuela. American Journal of Physical Anthropology 127: 361-369.

22. Marrero A, Silva-Junior W, Bravi C, Hutz M, Petzl-Erler M, et al. (2007) Demographic and evolutionary trajectories of the Guarani and Kaingang natives of Brazil. American Journal of Physical Anthropology 132: 301-310.

23. Ward RH, Salzno FM, Bonatto SL, Hutz MH, Coimbra CEA, et al. (1996) Mitochondrial DNA polymorphism is three Brazilian Indian Tribes. American Journal of Human Biology 8: 317-323.

24. Dornelles C, Battilana J, Fagundes N, Freitas L, Bonatto S, et al. (2004) Mitochondrial DNA and Alu insertions in a genetically peculiar population: the Ayoreo Indians of Bolivia and Paraguay. American Journal of Humam Biology 16: 479-488.

25. Schmitt R, Bonatto S, Freitas L, Muschner V, Kill K, et al. (2004) Extremely limited mitochondrial DNA variation among the Aché Natives of Paraguay. Annals of Human Biology 31: 87-94.
