# Supplementary material for: The Andean Adaptive Toolkit to Counteract High Altitude Maladaptation: Genome-Wide and Phenotypic Analysis of the Collas
Source: PLoS One. 2014 Mar 31;9(3):e93314. doi: 10.1371/journal.pone.0093314 (PMC3970967; doi:10.1371/journal.pone.0093314)
Supplement: Table S4 — mtDNA haplotypes and haplogroup assignment of Collas and Wichí. (DOCX) [file pone.0093314.s009.docx]

Table S4. mtDNA haplotypes and haplogroup assignment of Collas and Wichí.

| **Sample** | **Group** | **A** | **B** | **C** | **D** | **Haplotype^a^** | **Missing mutations** |
| --- | --- | --- | --- | --- | --- | --- | --- |
| S1 | Colla | A2*? |  |  |  | 16234, 16256, **16290,** **16319, 16362** | A2: 16111 |
| S2 | Colla |  |  | C1 |  | 16037, **16298, 16325, 16327** |  |
| S6 | Colla |  | B2 |  |  | **16183, 16189, 16217, 16223** |  |
| S9 | Colla |  | B2 |  |  | **16183, 16189, 16217, 16223** |  |
| S12 | Colla |  | B2 |  |  | **16183, 16189, 16217, 16223,** 16294 |  |
| S23 | Colla |  | B2? |  |  | 16145,16156, 16157, **16189, 16217, 16223** | B2: 16183 |
| S25 | Colla |  | B2 |  |  | **16183,** 16188, **16189, 16217, 16223** |  |
| S27 | Colla |  |  |  | D1 | 16293, **16325,16362** |  |
| S29 | Colla |  | B2 |  |  | **16183, 16189, 16217, 16223,** 16319, 16362 |  |
| S32 | Colla |  | B2 |  |  | **16183,** 16188, **16189, 16217, 16223** |  |
| S41 | Colla |  |  |  | D1 | 16189, 16194, 16195, **16325, 16362** |  |
| S42 | Colla | A2* |  |  |  | **16111, 16290, 16319, 16362** |  |
| S43 | Colla |  |  |  | D1 | **16325, 16362** |  |
| S47 | Colla | A2*? |  |  |  | 16234 R, 16256, **16290, 16319, 16362** | A2: 16111 |
| T1 | Colla |  | B2 |  |  | 16129, **16183, 16189, 16217, 16223,** 16309 |  |
| T2 | Colla |  | B2 |  |  | **16183, 16189, 16217, 16223,** 16294 |  |
| T4 | Colla |  | B2 |  |  | 16093, **16183, 16189, 16217, 16223** |  |
| T5 | Colla |  | B2 |  |  | 16124, **16183, 16189, 16217, 16223,** 16294 |  |
| T7 | Colla |  | B2 |  |  | **16183, 16189, 16217, 16223** |  |
| T11 | Colla |  | B2 |  |  | **16183, 16189, 16217, 16223,** 16289 |  |
| T12 | Colla | A2*? |  |  |  | **16111, 16319, 16362** | A: 16290 |
| T13 | Colla | A2*? |  |  |  | 16189, 16260, 16288, **16290, 16319, 16362** | A2: 16111 |
| O7 | Colla | A2*? |  |  |  | 16189, 16260, 16288, **16290, 16319, 16362** | A2: 16111 |
| E2 | Wichí |  | B2 |  |  | 16182, **16183**, 16188, **16189, 16217, 16223** |  |
| E3 | Wichí |  | B2 |  |  | 16182, **16183**, 16188, **16189, 16217, 16223** |  |
| E6 | Wichí |  |  |  | D1 | 16174, 16263, **16325,** 16361, **16362**, (16384 R), 16390 |  |
| E10 | Wichí |  | B2 |  |  | 16182, **16183, 16189, 16217, 16223** |  |
| E12 | Wichí |  | B2 |  |  | 16182, **16183, 16189**, 16198, **16217, 16223** |  |
| E13 | Wichí |  | B2 |  |  | 16182, **16183, 16189**, 16198, **16217, 16223** |  |
| E14 | Wichí |  |  |  | D1 | **16325, 16362** |  |
| DE20 | Wichí |  | B2 |  |  | 16140, 16182, **16183, 16189, 16217, 16223** |  |
| DE23 | Wichí |  |  |  | D1 | **16325, 16362** |  |
| DE24 | Wichí |  | B2 |  |  | 16182, **16183, 16189,** 16198, **16217, 16223** |  |
| DE27 | Wichí | A2* |  |  |  | 16051, **16111, 16290, 16319, 16362** |  |
| DE29 | Wichí | A2* |  |  |  | 16051, **16111, 16290, 16319, 16362** |  |
| C30 | Wichí |  |  |  | D1 | 16174, 16263, **16325,** 16361, **16362,** 16390 |  |
| C31 | Wichí |  | B2 |  |  | 16102, 16182, **16183, 16189, 16217, 16223** |  |
| C32 | Wichí |  |  |  | D1 | 16174, 16263, **16325,** 16361, **16362,** 16390 |  |
| MC34 | Wichí |  | B2 |  |  | 16182, **16183, 16189, 16217, 16223,** 16261, |  |
| MC38 | Wichí |  | B2 |  |  | 16182, **16183, 16189, 16217, 16223** |  |
| MC39 | Wichí |  | B2 |  |  | 16182, **16183, 16189,** 16198, **16217, 16223** |  |
| MC40 | Wichí |  |  |  | D1 | 16174, 16263, **16325**, 16361, **16362,** 16390 |  |
| MC41 | Wichí | A2* |  |  |  | 16051, **16111,** 16270 S, **16290, 16319, 16362** |  |

^a^L3 was used as an ancestral sequence to assign haplotypes; bold: haplogroup defining mutations
