# Supplementary material for: The Andean Adaptive Toolkit to Counteract High Altitude Maladaptation: Genome-Wide and Phenotypic Analysis of the Collas
Source: PLoS One. 2014 Mar 31;9(3):e93314. doi: 10.1371/journal.pone.0093314 (PMC3970967; doi:10.1371/journal.pone.0093314)
Supplement: Table S6 — Genes of interest in the 1 Mb region around VEGFB . (DOCX) [file pone.0093314.s011.docx]

Table S6. Genes of interest in the 1 Mb region around *VEGFB*.

| **Gene** | **Start** | **End** | **Name** | **Function** |
| --- | --- | --- | --- | --- |
| *MARK2* | 63606477 | 63607032 | Serine/threonine protein kinase EMK | Cardiovascular phenotype related |
| *COX8A* | 63742079 | 63742266 | Cytochrome c oxidase subunit VIII | Terminal enzyme of the respiratory chain |
| *STIP1* | 63952744 | 63972020 | HSP70/HSP90-organising protein | Mediates association of chaperones HSP70 and HSP90; HSP90 stabilises eNOS and HIF-1 |
| *FERMT3* | 63974150 | 63991363 | Fermitin family homolog 3 | Plays a central role in cell adhesion in haematopoietic cells |
| *VEGFB* | 64002010 | 64006259 | Vascular endothelial growth factor β | Growth factor for endothelial cells, predominantly expressed in the heart |
| *BAD* | 64037300 | 64052176 | BCL2-associated agonist of cell death | Positive regulation of cell apoptosis, hypoxia responsive |
| *KCNK4* | 64058774 | 64072241 | Potassium channel subfamily K member 4 | Potassium channel, ischemia related |
| *ESRRA* | 64073044 | 64084215 | Nuclear receptor subfamily 3 group B member 1 | Closely related to oestrogen receptor; oestrogen is involved in skeletal growth and cardiovascular functioning |
| *PRDX5* | 64085560 | 64089283 | Peroxisomal antioxidant enzyme | Reduces hydrogen peroxide |
| *SLC22A12* | 64358113 | 64369820 | Solute carrier family 22 member 12 | Required for efficient urate re-absorption in the kidney, regulates blood urate levels |
| *PYGM* | 64513861 | 64528187 | Phosphorylase, glycogen | Allosteric enzyme in carbohydrate metabolism |
