# Supplementary material for: The Andean Adaptive Toolkit to Counteract High Altitude Maladaptation: Genome-Wide and Phenotypic Analysis of the Collas
Source: PLoS One. 2014 Mar 31;9(3):e93314. doi: 10.1371/journal.pone.0093314 (PMC3970967; doi:10.1371/journal.pone.0093314)
Supplement: Table S7 — Candidate genes in the top 1% of PBS results in Collas. (DOCX) [file pone.0093314.s012.docx]

Table S7. Candidate genes in the top 1% of PBS results in Collas.

| **Rank^a^** | **Gene** | **Name** | **Function** | **Hypoxia/ arsenic association** | **Window PBS _MAX_:**  **Gene PBS _MAX_** |
| --- | --- | --- | --- | --- | --- |
| 2 | *CBS* | Serine sulfhydrase | Bradykinin receptor involved in NO mediated vasodilation; associated with CBF | Cellular response to hypoxia | 1.005:  1.005 |
| 52 | *PRKG1* | Protein kinase, cGMP-dependent | Key mediator NO pathway, regulates platelet activation and adhesion, smooth muscle contraction, cardiac function | NO stimulates guanylate cyclase | 0.693:  0.693 |
| 137 | *STC2* | Stanniocalcin 2 | Bone and skeletal muscle growth; HIF-1 activated, protects cells from apoptosis in hypoxia | Cellular response to hypoxia | 0.583:  41 kb downstream  0.050 |
| 172 | *FOXO1* | Forkhead box protein O1A | Transcription factor: main target of insulin signalling; regulates metabolic homeostasis in response to oxidative stress | Cellular response to ROS | 0.558:  0.558 |
| 190 | *UBE2D3* | Ubiquitin-protein ligase D3 | E2 ubiquitin-conjugating enzyme, targets p53 and EGFR among others | Cellular response to hypoxia | 0.548:  0.548 |
| 198 | *SOD1* | Superoxide dismutase 1 | Convert superoxide radicals to molecular oxygen and hydrogen peroxide | Cellular response to ROS | 0.545:  38 kb downstream  0.208 |
| 202 | *GNGT1* | Guanine nucleotide-binding protein G(T1) | GMPase, mediates activation by rhodopsin of a cyclic GTP-specific phosphodiesterase | Cellular response to hypoxia | 0.543:  0.543 |

^a^Lower ranks are reported for PBS as window size was halved compared to other tests.
