# Supplementary material for: The Andean Adaptive Toolkit to Counteract High Altitude Maladaptation: Genome-Wide and Phenotypic Analysis of the Collas
Source: PLoS One. 2014 Mar 31;9(3):e93314. doi: 10.1371/journal.pone.0093314 (PMC3970967; doi:10.1371/journal.pone.0093314)
Supplement: Table S8 — Genes detected with more than one selection test in Collas. (DOCX) [file pone.0093314.s013.docx]

Table S8. Genes detected with more than one selection test in Collas.

| **Gene** | ***F*_ST_** | **PBS** | **iHS** | **XP-EHH** |
| --- | --- | --- | --- | --- |
| *AS3MT* | x | x |  |  |
| *ASH1L* | x | x |  |  |
| *C10orf26* | x | x |  |  |
| *C10orf32* | x | x |  |  |
| *CCT4* | x | x |  |  |
| *CLK2* | x | x |  |  |
| *CNNM2* | x | x |  |  |
| *CYP17A1* | x | x |  |  |
| ***ELTD1*** | x | x |  | x |
| *FAM189B* | x | x |  |  |
| *FDPS* | x | x |  |  |
| *GRHL1* | x | x |  |  |
| *HCN3* | x | x |  |  |
| *HDLBP* | x | x |  |  |
| *MSTO1* | x | x |  |  |
| ***PDSS1*** | x | x | x |  |
| *PKLR* | x | x |  |  |
| *RUSC1* | x | x |  |  |
| *SCAMP3* | x | x |  |  |
| ***SUCLG2*** | x | x |  | x |
| *TACR3* | x | x |  |  |
| *TAF1B* | x | x |  |  |
| *ZC3H11A* | x | x |  | x |
| *RP11-223D18.1* | x |  |  | x |
| *snoU13* | x |  |  | x |
| *U4* | x |  |  | x |
| *U6* | x |  |  | x |
| *ABI1* |  | x | x |  |
| *ADCY4* |  | x | x |  |
| ***AP1S1*** |  | x | x | x |
| *AP4M1* |  | x | x |  |
| *C14orf21* |  | x | x |  |
| *CHKA* |  | x | x |  |
| *CIDEB* |  | x | x |  |
| *COPS6* |  | x | x |  |
| *DHRS1* |  | x | x |  |
| *GMPR2* |  | x | x |  |
| *LHFP* |  | x | x |  |
| *LTB4R* |  | x | x |  |
| *LTB4R2* |  | x | x |  |
| *MCM7* |  | x | x |  |
| ***MUC17*** |  | x | x | x |
| *NDUFS8* |  | x | x |  |
| *NEDD8* |  | x | x |  |
| *NEDD8-MDP1* |  | x | x |  |
| *PKNOX1* |  | x | x |  |
| *RABGGTA* |  | x | x |  |
| ***SERPINE1*** |  | x | x | x |
| *STC2* |  | x | x |  |
| *TCIRG1* |  | x | x |  |
| *TGM1* |  | x | x |  |
| *TINF2* |  | x | x |  |
| ***TRIM56*** |  | x | x | x |
| *ZKSCAN1* |  | x | x |  |
| *ZNF3* |  | x | x |  |
| *ZSCAN21* |  | x | x |  |
| *AC084082.3* |  | x |  | x |
| *ACY3* |  | x |  | x |
| *ALDH3B2* |  | x |  | x |
| *ATG2B* |  | x |  | x |
| *ATP2B4* |  | x |  | x |
| *BDKRB1* |  | x |  | x |
| *BDKRB2* |  | x |  | x |
| *C11orf86* |  | x |  | x |
| *C1R* |  | x |  | x |
| *C1RL* |  | x |  | x |
| *CACNA1B* |  | x |  | x |
| *CLSTN3* |  | x |  | x |
| *CRH* |  | x |  | x |
| *CSF3R* |  | x |  | x |
| *DNAJC5B* |  | x |  | x |
| *FREM2* |  | x |  | x |
| *HFM1* |  | x |  | x |
| *IGF1* |  | x |  | x |
| *MRPS15* |  | x |  | x |
| *OSCP1* |  | x |  | x |
| *PC* |  | x |  | x |
| *RAET1E* |  | x |  | x |
| *RAET1G* |  | x |  | x |
| *RBP5* |  | x |  | x |
| *RP11-404P21.6* |  | x |  | x |
| *RP11-404P21.8* |  | x |  | x |
| *SYT12* |  | x |  | x |
| *TBX10* |  | x |  | x |
| *TRIM55* |  | x |  | x |
| *ULBP1* |  | x |  | x |
| *ULBP2* |  | x |  | x |
| *DNAJC4* |  |  | x | x |
| *FERMT3* |  |  | x | x |
| *FLRT1* |  |  | x | x |
| *HAND2* |  |  | x | x |
| *MACROD1* |  |  | x | x |
| *MORF4* |  |  | x | x |
| *MUC12* |  |  | x | x |
| *MUC3A* |  |  | x | x |
| *NUDT22* |  |  | x | x |
| *PTGFR* |  |  | x | x |
| *RANP6* |  |  | x | x |
| *RP11-21A7A.2* |  |  | x | x |
| *RP11-21A7A.3* |  |  | x | x |
| *RP11-21A7A.4* |  |  | x | x |
| *RP11-395B7.2* |  |  | x | x |
| *RP11-395B7.4* |  |  | x | x |
| *RP11-471J12.1* |  |  | x | x |
| *RP11-783K16.14* |  |  | x | x |
| *SNORA43* |  |  | x | x |
| *STIP1* |  |  | x | x |
| *TRPT1* |  |  | x | x |

Bold: genes detected with three tests
