# Supplementary material for: The Andean Adaptive Toolkit to Counteract High Altitude Maladaptation: Genome-Wide and Phenotypic Analysis of the Collas
Source: PLoS One. 2014 Mar 31;9(3):e93314. doi: 10.1371/journal.pone.0093314 (PMC3970967; doi:10.1371/journal.pone.0093314)
Supplement: Table S9 — GO terms enrichment of the top 1% iHS windows in Collas with EASE-score <0.01. (DOCX) [file pone.0093314.s014.docx]

Table S9. GO terms enrichment of the top 1% iHS windows in Collas with EASE-score <0.01.

| **Category** | **EASE -score** | **GO term** | **Enriched genes** |
| --- | --- | --- | --- |
| **Aging** | 0.0068 | regulation of telomerase activity | 3/8 |
| **Apoptosis** | 0.0013 | negative regulation of apoptotic process | 15/430 |
|  | 0.0014 | negative regulation of programmed cell death | 15/435 |
|  | 0.0020 | negative regulation of cell death | 15/452 |
|  | 0.0025 | anti-apoptosis | 9/179 |
|  | 0.0077 | regulation of apoptotic process | 20/820 |
|  | 0.0088 | regulation of programmed cell death | 20/831 |
| **Cell proliferation/ development** | 0.0006 | regulation of cell proliferation | 23/828 |
|  | 0.0020 | negative regulation of cell proliferation | 14/401 |
|  | 0.0045 | cell cycle phase | 12/338 |
|  | 0.0056 | regulation of cell cycle | 15/507 |
|  | 0.0087 | cell cycle | 12/371 |
|  | 0.0090 | regulation of anatomical structure morphogenesis | 13/426 |
|  | 0.0091 | regulation of cell cycle arrest | 8/178 |
|  | 0.0099 | tissue development | 12/378 |
| **DNA regulation** | 0.0013 | sequence-specific DNA binding transcription factor activity | 19/644 |
|  | 0.0014 | nucleic acid binding transcription factor activity | 19/646 |
|  | 0.0014 | regulation of chromosome organization | 6/ 60 |
|  | 0.0014 | regulatory region DNA binding | 11/245 |
|  | 0.0014 | regulatory region nucleic acid binding | 11/245 |
|  | 0.0018 | regulation of transcription, DNA-dependent | 34/1611 |
|  | 0.0021 | chromatin modification | 11/260 |
|  | 0.0040 | transcription regulatory region DNA binding | 10/238 |
|  | 0.0049 | histone modification | 8/158 |
|  | 0.0051 | covalent chromatin modification | 8/159 |
|  | 0.0058 | positive regulation of histone modification | 4/26 |
|  | 0.0060 | sequence-specific DNA binding | 14/457 |
|  | 0.0077 | regulation of gene expression | 35/1823 |
| **General** | 0.0001 | transferase activity | 30/1137 |
|  | 0.0001 | chromosome organization | 16/391 |
|  | 0.0004 | transferase activity, transferring acyl groups | 10/167 |
|  | 0.0004 | N-acyltransferase activity | 7/70 |
|  | 0.0005 | chromatin organization | 13/301 |
|  | 0.0009 | positive regulation of chromosome organization | 5/30 |
|  | 0.0009 | multi-organism process | 17/517 |
|  | 0.0011 | N-acetyltransferase activity | 6/ 57 |
|  | 0.0011 | catalytic activity | 49/2689 |
|  | 0.0015 | organelle organization | 26/1069 |
|  | 0.0016 | regulation of cell adhesion | 10/207 |
|  | 0.0017 | transferase activity, transferring acyl groups other than amino-acyl groups | 8/130 |
|  | 0.0018 | macromolecule modification | 29/1275 |
|  | 0.0019 | cell cycle process | 17/555 |
|  | 0.002 | regulation of RNA biosynthetic process | 34/1619 |
|  | 0.0022 | protein modification process | 28/1223 |
|  | 0.0023 | protein heterodimerization activity | 10/219 |
|  | 0.0024 | acetyltransferase activity | 6/69 |
|  | 0.0027 | enzyme regulator activity | 18/630 |
|  | 0.0031 | positive regulation of nucleobase-containing compound metabolic process | 21/813 |
|  | 0.0032 | negative regulation of biological process | 36/1805 |
| **General** | 0.0044 | DNA metabolic process | 14/440 |
|  | 0.0047 | enzyme activator activity | 11/291 |
|  | 0.0053 | cellular response to cytokine stimulus | 10/249 |
|  | 0.0056 | regulation of cellular macromolecule biosynthetic process | 34/1717 |
|  | 0.0061 | positive regulation of cellular biosynthetic process | 21/863 |
|  | 0.0062 | negative regulation of nucleobase-containing compound metabolic process | 17/626 |
|  | 0.0069 | positive regulation of biosynthetic process | 21/873 |
|  | 0.0078 | regulation of macromolecule biosynthetic process | 34/1752 |
|  | 0.0085 | macromolecular complex | 37/1978 |
|  | 0.0089 | negative regulation of developmental process | 12/372 |
|  | 0.0090 | transferase activity, transferring phosphorus-containing groups | 17/652 |
|  | 0.0091 | DNA binding | 27/1284 |
|  | 0.0092 | peptidyl-amino acid modification | 11/322 |
|  | 0.0100 | response to cytokine stimulus | 11/326 |
| **Heart** | 0.0005 | cardiac ventricle formation | 4/10 |
|  | 0.0007 | cardiac chamber formation | 4/11 |
| **Immune response** | 0.0015 | response to virus | 8/126 |
|  | 0.0064 | cellular response to interleukin-1 | 4/27 |
|  | 0.0075 | response to other organism | 9/216 |
| **Ion related** | 0.0049 | ion transmembrane transport | 8/158 |
| **Metabolism** | 0.0011 | carbohydrate metabolic process | 16/473 |
|  | 0.0011 | catabolic process | 25/982 |
|  | 0.0011 | regulation of hydrolase activity | 18/580 |
|  | 0.0014 | positive regulation of hydrolase activity | 14/384 |
|  | 0.0018 | macromolecule glycosylation | 9/170 |
|  | 0.0018 | protein glycosylation | 9/170 |
|  | 0.0018 | glycosylation | 9/170 |
|  | 0.0032 | cellular carbohydrate metabolic process | 13/372 |
|  | 0.0055 | cellular protein metabolic process | 30/1439 |
|  | 0.0065 | regulation of nucleobase-containing compound metabolic process | 36/1877 |
|  | 0.0067 | hydrolase activity | 29/1389 |
|  | 0.0075 | protein O-linked glycosylation | 5/57 |
|  | 0.0097 | protein metabolic process | 33/1705 |
| **Morphogenesis** | 0.0063 | cartilage development | 5/54 |
| **Nitrogen** | 0.0037 | positive regulation of nitrogen compound metabolic process | 21/827 |
|  | 0.0072 | negative regulation of nitrogen compound metabolic process | 17/636 |
|  | 0.0089 | regulation of nitrogen compound metabolic process |  |
| **RNA regulation** | 0.0029 | regulation of RNA metabolic process | 34/1654 |
|  | 0.0085 | negative regulation of transcription from RNA polymerase II promoter | 11/318 |
|  | 0.0087 | regulation of transcription from RNA polymerase II promoter | 19/769 |
| **RNA regulation** | 0.0095 | positive regulation of RNA metabolic process | 18/715 |
| **Stress response** | 0.0007 | cellular response to stress | 20/663 |
|  | 0.0010 | response to DNA damage stimulus | 14/372 |
|  | 0.0023 | cellular response to abiotic stimulus | 7/101 |
|  | 0.0024 | response to oxidative stress | 9/178 |
|  | 0.0085 | response to stress | 28/1345 |
| **Structure** | 0.0000 | membrane part | 55/2829 |
|  | 0.0001 | endoplasmic reticulum | 19/494 |
|  | 0.0003 | integral to membrane | 47/2397 |
|  | 0.0003 | chromosomal part | 14/323 |
|  | 0.0004 | intrinsic to membrane | 47/2443 |
|  | 0.0005 | nucleoplasm part | 18/543 |
|  | 0.0005 | intrinsic to plasma membrane | 22/761 |
|  | 0.0007 | protein complex | 37/1743 |
|  | 0.0009 | integral to plasma membrane | 21/733 |
|  | 0.0013 | apical plasma membrane | 9/161 |
|  | 0.0014 | plasma membrane part | 29/1252 |
|  | 0.0022 | chromatin | 8/136 |
|  | 0.0056 | perinuclear region of cytoplasm | 12/349 |
|  | 0.0058 | nuclear chromosome part | 8/163 |
|  | 0.0064 | insoluble fraction | 18/687 |
|  | 0.0079 | organelle part | 51/3061 |
|  | 0.0091 | nuclear part | 33/1698 |
|  | 0.0097 | nucleoplasm | 16/599 |
|  | 0.0099 | cohesin complex | 3/10 |
