# Supplementary material for: The Andean Adaptive Toolkit to Counteract High Altitude Maladaptation: Genome-Wide and Phenotypic Analysis of the Collas
Source: PLoS One. 2014 Mar 31;9(3):e93314. doi: 10.1371/journal.pone.0093314 (PMC3970967; doi:10.1371/journal.pone.0093314)
Supplement: Table S10 — Enriched GO terms in the XP-EHH top 1% in Collas. (DOCX) [file pone.0093314.s015.docx]

Table S10. Enriched GO terms in the XP-EHH top 1% in Collas.

| **Category** | **EASE-score** | **GO term** | **Enriched genes** |
| --- | --- | --- | --- |
| **General** | 0.0020 | system process | 23/855 |
|  | 0.0036 | binding | 88/5815 |
|  | 0.0042 | cellular biosynthetic process | 47/2464 |
|  | 0.0058 | cellular macromolecule biosynthetic process | 38/1882 |
|  | 0.0069 | G-protein coupled receptor signalling pathway | 15/497 |
|  | 0.0071 | biosynthetic process | 47/2525 |
|  | 0.0074 | macromolecule biosynthetic process | 38/1909 |
|  | 0.0081 | secretion | 13/402 |
| **Neuron** | 0.0012 | neurological system process | 18/557 |
|  | 0.0023 | neuron differentiation | 9/169 |
|  | 0.0053 | sensory perception | 13/380 |
|  | 0.0071 | cell differentiation in spinal cord | 4/27 |
| **Structure** | 0.0078 | extracellular region part | 22/899 |
