# Supplementary material for: The Andean Adaptive Toolkit to Counteract High Altitude Maladaptation: Genome-Wide and Phenotypic Analysis of the Collas
Source: PLoS One. 2014 Mar 31;9(3):e93314. doi: 10.1371/journal.pone.0093314 (PMC3970967; doi:10.1371/journal.pone.0093314)
Supplement: Table S11 — GO term enrichment of PBS genes in the top 1% in Collas. (DOCX) [file pone.0093314.s016.docx]

Table S11. GO term enrichment of PBS genes in the top 1% in Collas.

| **Category** | **EASE-score** | **GO term** | **Fold enrichment** |
| --- | --- | --- | --- |
| **General** | 0.0048 | phosphatase regulator activity | 12 |
| **Ion related** | 0.0012 | calcium ion binding | 3 |
|  | 0.0014 | ion transport | 3 |
|  | 0.0024 | ion channel complex | 5 |
|  | 0.0028 | gated channel activity | 4 |
|  | 0.0038 | metal ion transmembrane transporter activity | 4 |
|  | 0.0067 | voltage-gated ion channel activity | 5 |
|  | 0.0067 | voltage-gated channel activity | 5 |
|  | 0.0090 | ion channel activity | 3 |
| **Neuron** | 0.0009 | transmission of nerve impulse | 4 |
|  | 0.0034 | neuron development | 4 |
