# Supplementary material for: The Andean Adaptive Toolkit to Counteract High Altitude Maladaptation: Genome-Wide and Phenotypic Analysis of the Collas
Source: PLoS One. 2014 Mar 31;9(3):e93314. doi: 10.1371/journal.pone.0093314 (PMC3970967; doi:10.1371/journal.pone.0093314)
Supplement: Table S12 — Hypoxia genes identified in this study and in other HA studies. (DOCX) [file pone.0093314.s017.docx]

Table S12. Hypoxia genes identified in the top 1% of this study and in other HA studies.

| **Population** | **Gene** | **Test (rank)** | **Other study^a^** | **Function** |
| --- | --- | --- | --- | --- |
| Collas | *CYP17A1* | *F*_ST_ (38) | T: Simonson *et al* [[1](#_ENREF_1)] | Cytochrome p450 |
|  | *FOXO1* | PBS (172) | T: Wang *et al* [[2](#_ENREF_2)] | Cellular response to ROS |
|  | *PKLR* | PBS (240)  *F*_ST_ (13) | T: Yi *et al* [[3](#_ENREF_3)] | Erythrocyte maintenance |
|  | *PRKAA2* | XP-EHH (31) | A: Bigham *et al* [[4](#_ENREF_4),[5](#_ENREF_5)] | Myocardial energy balance during ischemia; HIF pathway gene |
|  | *SYNJ2* | iHS (38) | E: Scheinfeldt *et al* [[6](#_ENREF_6)] | Phosphatidyl-inositol pathway |

^a^A= Andean, T= Tibetan, E=Ethiopian

**Supplemental References**

1. Simonson TS, Yang Y, Huff CD, Yun H, Qin G, et al. (2010) Genetic evidence for high-altitude adaptation in Tibet. Science 329: 72-75.

2. Wang B, Zhang YB, Zhang F, Lin H, Wang X, et al. (2011) On the origin of Tibetans and their genetic basis in adapting high-altitude environments. PLoS One 6: e17002.

3. Yi X, Liang Y, Huerta-Sanchez E, Jin X, Cuo ZX, et al. (2010) Sequencing of 50 human exomes reveals adaptation to high altitude. Science 329: 75-78.

4. Bigham A, Bauchet M, Pinto D, Mao X, Akey JM, et al. (2010) Identifying signatures of natural selection in Tibetan and Andean populations using dense genome scan data. PLoS Genet 6: e1001116.

5. Bigham AW, Mao X, Mei R, Brutsaert T, Wilson MJ, et al. (2009) Identifying positive selection candidate loci for high-altitude adaptation in Andean populations. Hum Genomics 4: 79-90.

6. Scheinfeldt LB, Soi S, Thompson S, Ranciaro A, Meskel DW, et al. (2012) Genetic adaptation to high altitude in the Ethiopian highlands. Genome Biol 13: R1.
