# Supplementary material for: The Andean Adaptive Toolkit to Counteract High Altitude Maladaptation: Genome-Wide and Phenotypic Analysis of the Collas
Source: PLoS One. 2014 Mar 31;9(3):e93314. doi: 10.1371/journal.pone.0093314 (PMC3970967; doi:10.1371/journal.pone.0093314)
Supplement: Table S13 — Gene overlap in the top 5% of this study and Bigham et al [1],[2] and Zhou et al [3] . (DOCX) [file pone.0093314.s018.docx]

Table S13. Gene overlap in the top 5% of this study and Bigham *et al* [[1](#_ENREF_1),[2](#_ENREF_2)] and Zhou *et al* [[3](#_ENREF_3)].

| **Gene** | **iHS rank** | **XP-EHH rank** | ***F*_ST_ rank** | **PBS rank** |
| --- | --- | --- | --- | --- |
| *AGTRAP* |  |  |  | 344 |
| *ANP32D* |  | 606 |  |  |
| *ARID1B* | 205 |  |  |  |
| *ARNT2* |  |  | 385 |  |
| *ATP1A2* |  |  |  | 372 |
| *C12orf54* |  | 606 |  |  |
| *CDH1* |  | 662 |  |  |
| *CNNM1* | 456 |  |  |  |
| *EGLN2* | 152 |  |  |  |
| *IGFBP2* |  |  |  | 552 |
| ***PRKAA2*** |  | 31 |  | 1098 |
| *SATB1* | 194 |  |  |  |
| *SPRY2* |  | 638 |  | 669 |

Bold genes are found among the top 1% in this study of at least one test.

**Supplemental References**

1. Bigham A, Bauchet M, Pinto D, Mao X, Akey JM, et al. (2010) Identifying signatures of natural selection in Tibetan and Andean populations using dense genome scan data. PLoS Genet 6: e1001116.

2. Bigham AW, Mao X, Mei R, Brutsaert T, Wilson MJ, et al. (2009) Identifying positive selection candidate loci for high-altitude adaptation in Andean populations. Hum Genomics 4: 79-90.

3. Zhou D, Udpa N, Ronen R, Stobdan T, Liang J, et al. (2013) Whole-Genome Sequencing Uncovers the Genetic Basis of Chronic Mountain Sickness in Andean Highlanders. Am J Hum Genet.
